# Development and Validation of a Clinical Prediction Model for Sleep Disorders in the ICU: A Retrospective Cohort Study

**DOI:** 10.3389/fnins.2021.644845

**Published:** 2021-04-16

**Authors:** Yun Li, Lina Zhao, Chenyi Yang, Zhiqiang Yu, Jiannan Song, Qi Zhou, Xizhe Zhang, Jie Gao, Qiang Wang, Haiyun Wang

**Affiliations:** ^1^The Third Central Clinical College of Tianjin Medical University, Tianjin, China; ^2^Tianjin Key Laboratory of Extracorporeal Life Support for Critical Diseases, Artificial Cell Engineering Technology Research Center, Tianjin Institute of Hepatobiliary Disease, Tianjin, China; ^3^Department of Anesthesiology, Chifeng Municipal Hospital, Chifeng Clinical Medical College of Inner Mongolia Medical University, Chifeng, China; ^4^Emergency Department, Peking Union Medical College Hospital, Peking Union Medical College, Chinese Academy of Medical Sciences, Beijing, China; ^5^Department of Anesthesiology, The Third Central Hospital of Tianjin, The Third Central Clinical College of Tianjin Medical University, Nankai University Affinity the Third Central Hospital, Tianjin Key Laboratory of Extracorporeal Life Support for Critical Diseases, Artificial Cell Engineering Technology Research Center, Tianjin Institute of Hepatobiliary Disease, Tianjin, China; ^6^Department of Anesthesiology, Tianjin Central Hospital of Gynecology and Obstetrics, Tianjin Key Laboratory of Human Development and Reproductive Regulation, Tianjin, China

**Keywords:** MIMIC-III database, sleep disorders, risk, prediction model, ICU

## Abstract

**Background:**

Sleep disorders, the serious challenges faced by the intensive care unit (ICU) patients are important issues that need urgent attention. Despite some efforts to reduce sleep disorders with common risk-factor controlling, unidentified risk factors remain.

**Objectives:**

This study aimed to develop and validate a risk prediction model for sleep disorders in ICU adults.

**Methods:**

Data were retrieved from the MIMIC-III database. Matching analysis was used to match the patients with and without sleep disorders. A nomogram was developed based on the logistic regression, which was used to identify risk factors for sleep disorders. The calibration and discrimination of the nomogram were evaluated with the 1000 bootstrap resampling and receiver operating characteristic curve (ROC). Besides, the decision curve analysis (DCA) was applied to evaluate the clinical utility of the prediction model.

**Results:**

2,082 patients were included in the analysis, 80% of whom (*n* = 1,666) and the remaining 20% (*n* = 416) were divided into the training and validation sets. After the multivariate analysis, hemoglobin, diastolic blood pressure, respiratory rate, cardiovascular disease, and delirium were the independent risk predictors for sleep disorders. The nomogram showed high sensitivity and specificity of 75.6% and 72.9% in the ROC. The threshold probability of the net benefit was between 55% and 90% in the DCA.

**Conclusion:**

The model showed high performance in predicting sleep disorders in ICU adults, the good clinical utility of which may be a useful tool for providing clinical decision support to improve sleep quality in the ICU.

## Introduction

Sleep is an important part of maintaining the normal physiological and psychological state of human beings (Irwin, 2015). However, sleep disorders are common and severe in the intensive care unit (ICU) patients, with a prevalence of more than 50% (Bani Younis and Hayajneh, 2018), and even up to approximately 80% in severe cases (Little et al., 2012). 10-61% of the patients even had sleep problems over 6 months after discharge, which seriously affected the long-term recovery (Altman et al., 2017). Moreover, sleep disorders are associated with a wide range of dysfunctions, including cardio-cerebrovascular disease, disturbed metabolism, low immunity, and impaired cognition (Irwin, 2015; Nicholson et al., 2020).

As a special group, ICU patients have the feature of severity and complexity, coupled with special monitoring and intensive treatment, which make their sleep problems more likely to be ignored (Devlin et al., 2018). And the mechanisms leading to sleep disorders in ICU patients are complex and multivariate, with relevance to the environmental, pharmaceutical and medical factors, as well as their interactions (Kamdar et al., 2012). Although the criteria for diagnosis and assessment of sleep disorders are applicable, they also are complicated in the ICU due to the practical challenge and difficulty (Devlin et al., 2018; Telias and Wilcox, 2019). Unfortunately, no high-quality medication is recommended to improve the sleep quality of ICU patients (Kamdar et al., 2013). It is now known that light and noise of environmental factors severely interfere with the sleep-waking cycle (Bani Younis et al., 2019). But the eye mask and earplug of non-pharmacological interventions and melatonin can effectively ameliorate such patients (Demoule et al., 2017; Lewis et al., 2018; Telias and Wilcox, 2019). Despite 20% of medical procedures disrupt the sleep process, they only account for 7% of ICU patients with sleep disorders (Pisani et al., 2015). As a result, most other ICU patients with sleep disorders may not get the attention they deserve.

The sleep of ICU patients is generally disturbed, and the pattern of sleep disorders is apparently complicated (Kamdar et al., 2012; Boyko et al., 2017; Jolley et al., 2018). Specifically, non-specific sleep disorders, including their subtypes (e.g., insomnia, unspecified sleep apnea, sleep-wake circadian rhythm disorder, parasomnias, and other non-specific sleep problems) are common problems that affect ICU patients. Despite specific sleep disorders, including obstructive, central, and hypoventilation sleep-disordered breathing, central somnolence disorder, and sleep-related movement disorder, as well as idiopathic sleep disorders, also occur in ICU patients, they lack predictive value due to their specific etiology or low incidence.

Medical staff should pay attention to sleep problems in ICU patients (Devlin et al., 2018). Moreover, the research perspective of sleep disorders in the ICU also should shift from emphasizing intervention to focusing on risk prediction, especially the clinical modifiable risks. Our study aimed to develop a risk prediction model for non-specific sleep disorders in the ICU adults from the Medical Information Mart for Intensive Care III (MIMIC-III) database. Furthermore, identifying the potentially modifiable risk predictor was the focus of our study.

## Materials and Methods

### Data Source

The data of our study were retrieved and extracted from the MIMIC-III database. The MIMIC-III database was derived from detailed medical records of ICU patients in the Beth Israel Deaconess Medical Center between June 1, 2002 and October 31, 2012 (Johnson et al., 2016). There were more than 50,000 adult patients admitted to the ICU in the MIMIC-III database. The database included demographic information, vital signs, laboratory tests, imaging examinations, medications, disease codes, and other clinical data. And the data sets of all patients in the database were anonymous and publicly available.

### Patient Population

From the MIMIC-III database, we included adult patients of ICU admission with an essential information level, including the admission and discharge records. In addition, the duration of ICU stay for more than 24 h was also one of the inclusion criteria.

The exclusion criteria were as follows: (1) specific sleep disorders on discharge according to the International Classification of Diseases, Ninth Revision (ICD-9) codes, including sleep-disordered breathing (primary central sleep apnea, high altitude periodic breathing, idiopathic sleep-related non-obstructive alveolar hypoventilation, congenital central alveolar hypoventilation syndrome, etc.), central narcolepsy, sleep-related movement disorder (restless legs syndrome, periodic limb movement disorder, sleep-related leg cramps, sleep-related bruxism, etc.), and other specific sleep disorders (fatal familial insomnia, insomnia due to mental disorder, alcohol-induced sleep disorders, etc.), as well as idiopathic sleep disorders (Supplementary Material 1); (2) age < 18 years old.

## Outcome Assessment

The endpoint of our cohort was non-specific sleep disorders on discharge, which were as follows: insomnia; sleep apnea, unspecified; sleep disturbance, unspecified; lack of adequate sleep; excessive sleepiness; circadian rhythm sleep disorder; sleep-wake cycle disorder; parasomnias; and other non-specific sleep problems (Supplementary Material 2).

### Prediction Variables

The data sets of eligible patients, extracted from the MIMIC-III database, were used to screen out risk variables of sleep disorders. The covariates contained demographic information, types of ICU admission, vital signs, laboratory tests, comorbid diseases, illness score systems, medications, and medical procedures (Supplementary Materials 3, 4). Mean values of vital signs on the first 24 h of ICU admission and the first laboratory tests were recorded. According to the drug codes, medications included antibiotics, analgesic and sedative drugs, cardiovascular drugs, and corticosteroids during the hospitalization. Mechanical ventilation, an important medical treatment, was determined. We also assessed the severity of critical illness based on the Simplified Acute Physiology Score (SAPS II), Sequential Organ Failure Assessment (SOFA), and Glasgow Coma Scale (GCS).

### Statistical Analysis

Data distribution was tested using the Shapiro-Wilk test, and the results showed they were all non-normal. Continuous and categorical variables were expressed by the median (interquartile range, IQR) and the frequency (percentage), respectively. The non-parametric test (Mann-Whitney U test or Kruskal-Wallis test) was applied for the data, such as the age, vital signs, laboratory test, illness score system, and duration of ICU stay. The sex, type of ICU admission, comorbidity, medication, medical procedure, and hospital mortality were analyzed using the Pearson Chi-squared test. By the propensity matching analysis based on age and gender, case-control patients were matched to patients with sleep disorders in a 2:1 ratio.

The logistic regression model was used for univariate and multivariate analysis of sleep disorders. A nomogram was developed based on the results of the multivariate analysis. The calibration of the nomogram was used for internal validation by the 1000 bootstrap resampling procedure. The discrimination ability of the nomogram was quantified with the receiver operating characteristic curve (ROC). The clinical utility of the nomogram was assessed with the decision curve analysis (DCA).

We replaced any missing values of the included variables with multiple imputations (Supplementary Material 5). Statistical analysis was conducted with R software (version 3.4.3). Statistical significance was defined as *p* < 0.05.

**TABLE 1:** Characteristics of patients in the training and validation sets.

	**Primary Cohort**			**Validation Cohort**		
	**Sleep disorders Group, *n* = 545**	**Non-sleep Disorders group, *n* = 1121**	**P**	**Sleep disorders Group, *n* = 149**	**Non-sleep Disorders group, *n* = 267**	**P**
**Age [years, median (IQR)]**	62.1 (52.0–73.2)	62.4 (51.4–74.9)	0.835	61.6 (51.0–74.2)	61.6 (52.1–71.3)	0.926
**Sex [*n* (%)]**						
Female	203 (37.2)	445 (39.7)	0.336	54 (36.2)	99 (37.1)	0.865
Male	342 (62.8)	676 (60.3)		95 (63.8)	168 (62.9)	
**Type of ICU admission [*n* (%)]**			0.014			< 0.001
Emergency	415 (76.1)	897 (80.0)		118 (79.2)	214 (80.1)	
Elective	115 (21.1)	197 (36.1)		29 (19.5)	43 (16.1)	
Urgent	15 (2.8)	27 (2.4)		2 (1.3)	10 (3.7)	
**Vital signs [median (IQR)]**						
Heart rate(bpm)	101(89–103)	101 (89–105)	0.946	100 (89–114.5)	105 (91–115)	0.143
Diastolic blood pressure (mmHg)	44 (36.5–50)	44 (38–52)	0.025	46 (40–53.5)	44 (37–53)	0.142
Systolic blood pressure (mmHg)	90 (81–102)	92 (83–102)	0.113	94 (82.5–106.5)	91 (81–101)	0.157
Respiratory rate (bpm)	26 (23–31)	22 (26–39)	0.001	26 (23–29)	27 (23–30)	0.464
Temperature (°C)	37.4 (37–37.9)	37.4 (37–38)	0.786	37.4 (37–38.1)	37.5 (37–38)	0.237
SpO_2_ (%)	100 (99–100)	100 (100–100)	0.195	100 (99–100)	100 (100–100)	0.200
**Laboratory test [median (IQR)]**						
Alanine aminotransferase (IU/L)	31 (18–44.7)	30 (18.0–44.7)	0.095	32 (18–48.8)	25 (17–47)	0.389
Aspartate aminotransferase (IU/L)	36 (21–73.8)	34 (21–73.8)	0.530	37 (24–47.4)	29 (21-54)	0.876
Albumin (g/dL)	4.0 (2.0–4.6)	4.0 (1.0–4.3)	0.495	4.0 (1.0–4.3)	4.0 (1.0–4.3)	0.806
Creatinine (mg/dL)	1.0 (0.8–1.4)	1.0 (0.8–1.4)	0.055	1.0 (0.7–1.4)	1.0 (0.8–1.3)	0.141
Blood urea nitrogen (mg/dL)	20 (15–31)	19 (13–29)	0.091	20 (14–32)	18 (13–29)	0.092
Hemoglobin (g/dL)	10.5 (9.1–11.9)	10.3 (9.0–11.6)	0.041	10.7 (9.2–12.3)	10.0 (8.7–11.6)	0.810
Platelet (10^9^/L)	197 (140.5–253)	181 (129–242.5)	0.006	200 (160.5–269)	188 (128–247)	0.319
Partial thromboplastin time (s)	33.4 (27.7–42.6)	33.7 (27.9–42.6)	0.381	31.8 (26.4–42.6)	34.1 (28–42.6)	0.215
International normalized ratio	1.4 (1.2–1.6)	1.3 (1.2–1.6)	0.105	1.3 (1.1–1.6)	1.3 (1.2–1.6)	0.677
Prothrombin time (s)	15.0 (13.6–16.7)	14.9 (13.4–16.7)	0.146	14.4 (13.1–16.7)	14.7 (13.3–16.7)	0.291
White blood cell count (10^9^/L)	12.4 (8.7–16.3)	12.3 (9.0–16.5)	0.509	11.4 (8.3–14.5)	12.7 (9.7–17.1)	0.944
Sodium (mmol/L)	138 (135–140)	138 (135–140)	0.541	140 (138–142)	140 (137–142)	0.384
Potassium (mmol/L)	4.0 (3.6–4.3)	3.5 (3.9–4.2)	0.003	3.9 (3.6–4.2)	3.9 (3.5–4.3)	0.251
PH	7.42 (7.40–7.44)	7.42 (7.41–7.46)	0.014	7.31 (7.31–7.36)	7.31 (7.29–7.35)	0.274
Lactate (mmol/l)	3.0 (2.0–3.0)	3.0 (2.1–3.0)	0.973	3.0 (1.7–3.0)	3.0 (2.0–3.0)	0.385
Glucose (mg/dL)	105.9 (89–125)	103 (87–121)	0.021	106 (89–126)	106 (89–122)	0.548
**Comorbidity [n(%)]**						
Hypertension	333 (61.1)	656 (50.4)	0.314	90 (60.4)	141 (52.8)	0.135
Diabetes	161 (29.5)	326 (29.1)	0.846	44 (29.5)	65 (24.3)	0.249
Cardiovascular diseases	368 (67.5)	661 (59.0)	0.001	86 (57.7)	159 (59.6)	0.716
Chronic pulmonary disease	118 (21.7)	204 (18.2)	0.094	30 (20.1)	38 (14.2)	0.119
Liver disease	38 (7.0)	81 (7.2)	0.851	9 (6.0)	18 (6.7)	0.781
Kidney disease	216 (39.6)	422 (37.6)	0.434	56 (37.6)	92 (34.5)	0.523
Coagulation dysfunction	388 (71.2)	731 (65.5)	< 0.001	33 (22.1)	92 (34.5)	0.009
Anemias	207 (38.0)	369 (32.9)	0.041	59 (39.6)	78 (29.2)	0.031
Delirium	41 (7.5)	44 (3.9)	< 0.001	12 (8.1)	10 (3.7)	0.060
Cognitive dysfunction	5 (0.9)	13 (1.2)	0.654	1 (0.7)	2 (0.75)	0.928
**Medication [n (%)]**						
Antibiotics (%)						
Macrolides	14 (2.6)	25 (2.2)	0.668	3 (2.0)	7 (2.6)	0.698
Aminoglycosides	6 (1.1)	15 (1.3)	0.683	2 (1.3)	3 (1.1)	0.844
Quinolones	58 (10.6)	132 (11.8)	0.495	16 (10.7)	32 (12.0)	0.703
Beta lactam antibiotics	49 (9.0)	130 (11.6)	0.107	8 (5.4)	33 (12.4)	0.022
Analgesic and sedative drugs						
Opioids	108 (19.8)	237 (21.1)	0.531	28 (18.8)	55 (20.6)	0.658
Midazolam	39 (7.2)	87 (7.8)	0.661	6 (4.0)	24 (9.0)	0.061
Propofol	66 (12.1)	141 (12.6)	0.786	15 (10.1)	36 (13.5)	0.308
Etomidate	5 (0.9)	10 (0.9)	0.959	1 (0.7)	2 (0.75)	0.928
Dexmedetomidine	7 (1.3)	6 (0.5)	0.103	2 (1.3)	3 (1.1)	0.844
Benzodiazepines	66 (12.1)	152 (13.6)	0.411	18 (12.1)	27 (10.1)	0.535
Cardiovascular drugs (%)						
Norepinephrine	57 (10.5)	99 (8.8)	0.285	12 (8.1)	20 (7.5)	0.836
Epinephrine	13 (2.4)	35 (3.1)	0.399	9 (6.0)	13 (4.9)	0.609
Dobutamine	11 (2.0)	9 (0.8)	0.033	1 (0.7)	4 (1.5)	0.458
Dopamine	30 (5.6)	62 (5.5)	0.963	3 (2.0)	18 (6.7)	0.035
ß.blockers	89 (16.3)	180 (16.1)	0.887	24 (16.1)	27 (10.1)	0.074
Corticosteroids (%)	37 (6.8)	97 (8.7)	0.189	10 (6.7)	25 (9.4)	0.350
**Score system [median (IQR)]**						
SAPSII	31 (23–39)	31 (23–42)	0.224	30 (22.5–38.5)	33 (24–42)	0.216
SOFA	3 (2–5)	3 (2–5.5)	0.553	4 (1–5)	3 (2–5)	0.667
GCS	15 (14–15)	15 (14–15)	0.06	15 (14–15)	15 (14–15)	0.499
**Medical treatment**						
Mechanical ventilation [*n* (%)]	263 (48.3)	578 (51.6)	0.225	69 (46.3)	145 (54.3)	0.118
Renal replacement [n (%)]	10 (1.8)	25 (2.2)	0.593	3 (2.0)	8 (3.0)	0.549
duration of ICU stay [days, median (IQR)]	2.2 (1.3–4.2)	2.2 (1.2–4.3)	0.850	2.0 (1.1–3.3)	2.1 (1.2–4.1)	0.167
Hospital mortality [*n*(%)]	33(6.1)	139(12.4)	< 0.001	9(6.0)	33(12.4)	0.040

*IQR, interquartile range; SAPSII, simplified acute physiology score; SOFA, sequential organ failure assessment; GCS, glasgow coma scale; ICU, Intensive Care Unit. *p* < 0.05 means significant difference.*

## Results

### Patient Selection

**FIGURE 1:**
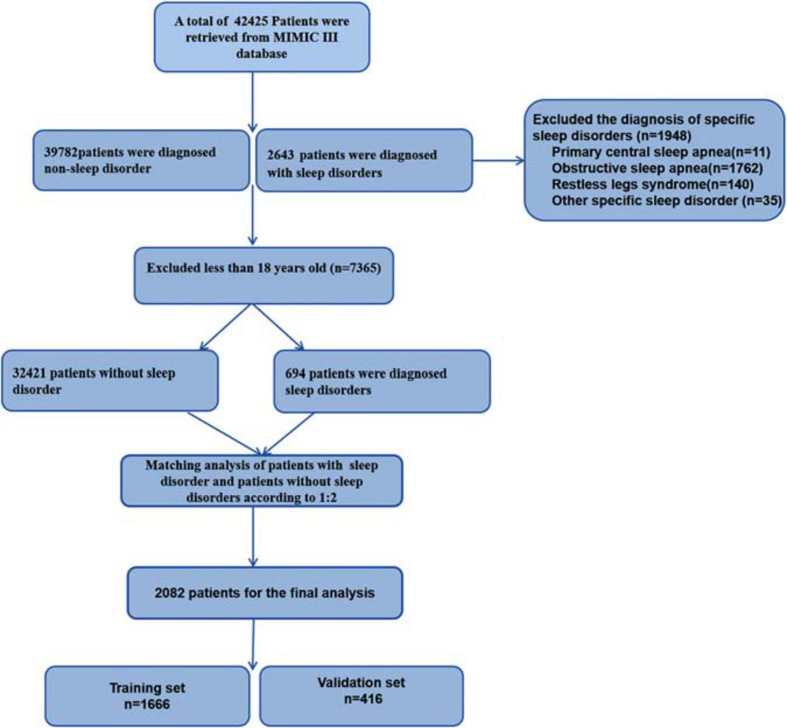
Flow chart of study cohort. MIMIC, Medical Information Mart for Intensive Care.

Of the 42,425 patients retrieved from the MIMIC-III database, 2,643 patients were diagnosed with sleep disorders, and 39,782 patients were not. After applying the exclusion criteria, 694 of the remaining 33,115 patients met the sleep-disorders criteria. 1,388, randomly drawn from the case-control group with 32,421 patients, were matched to the 694 patients with sleep disorders in a ratio of 2:1. Finally, 2,082 patients were randomly divided into the training set with 1,666 patients (80%) and the validation set with 416 patients (20%). The patient selection process is shown in Figure 1.

### Patient Characteristics

Clinical variables in the study population were analyzed by univariate logistic regression for sleep disorders. In terms of the type of ICU admission and hospital mortality, they were statistical differences in the training and validation sets, respectively. Diastolic blood pressure and respiratory rate of the patients with sleep disorders in the training set were lower than those without sleep disorders (36.5–50 vs 38–52 mmHg and 23–31 vs 26–39 bpm, respectively). There were statistical differences in the levels of hemoglobin (9.1–11.9 vs 9.0–11.6 g/dL), platelet (140.5–253 vs 129–242.5 10^9^/L), potassium (3.6–4.3 vs 3.9–4.2 mmol/L), PH (7.40–7.44 vs 7.41–7.46) and glucose (89–125 vs 87–121 mg/dL), and in the incidences of cardiovascular disease (67.5% vs 59.0%), coagulation dysfunction (71.2% vs 65.5%), Anemias (38.0% vs 32.9%), delirium (7.5% vs 3.9%), and dobutamine (2.0% vs 0.8%) of the patients in the training set. The two sets did not show any statistical difference in other major variables. Clinical characteristics of the study population are shown in Table 1.

### Multivariate Analysis of Risk Predictors to Sleep Disorders

The variables with *p* < 0.05 in the training set (Table 1) were taken as potential risk predictors for multivariate logistic regression procedure. In the multivariate analysis with results expressed as odds ratio (95% confidence interval), diastolic blood pressure (mmHg) [1.010 (1.001, 1.020)], respiratory rate (bpm) [0.975 (0.959, 0.991)], hemoglobin (g/dL) [0.938 (0.886, 0.992)], delirium [1.762 (1.110, 2.796)], and cardiovascular diseases [1.410 (1.130, 1.760)] were statistically independent predictors, which were associated with sleep disorders after adjusting for platelet, potassium, PH, glucose, coagulation dysfunction and anemias. The results of multivariate logistic regression analysis for sleep disorders are shown in Table 2.

**TABLE 2:** Multivariate logistic regression analysis of risk predictors to sleep disorders.

	**Multivariate analysis**			
	**OR**	**95.0% CI**		***p*-values**
		**Lower**	**Upper**	
Diastolic blood pressure (mmHg)	1.010	1.001	1.020	0.034
Respiratory rate (bpm)	0.975	0.959	0.991	0.002
Hemoglobin(g/dL)	0.938	0.886	0.992	0.025
Platelet (10^9^/L)	1.000	0.999	1.001	0.579
Potassium (mmol/L)	0.899	0.749	1.079	0.254
PH	2.465	0.513	11.846	0.260
Glucose (mg/dL)	0.997	0.994	1.000	0.77
Coagulation dysfunction	0.840	0.634	1.112	0.223
Delirium	1.762	1.110	2.796	0.016
Anemias	1.205	0.956	1.519	0.115
Cardiovascular diseases	1.410	1.130	1.760	0.002
Dobutamine	2.286	0.921	5.675	0.075

### Development of a Sleep-Disorder Prediction Nomogram

**FIGURE 2:**
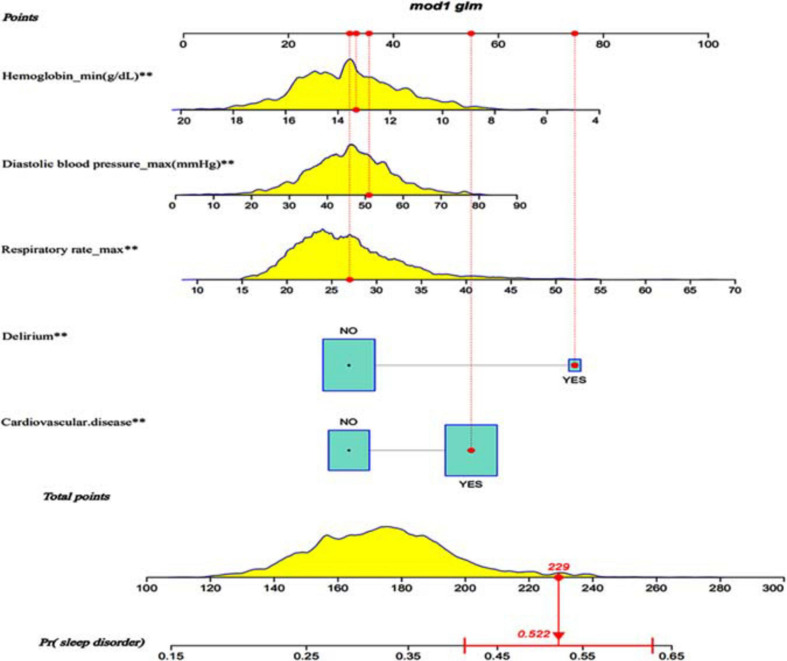
Visualization nomogram to predict the occurrence of sleep disorders. Fist a standard line marked with 0–100 points is drawn from the points axis. Then each of five predictors (Diastolic blood pressure, hemoglobin, respiratory rate, cardiovascular disease and delirium) corresponds to a parameter value by drawing a visualization line graph from the point axis. Finally, a line from the total-points axis, which is the sum of the points for each of the five predictors in the range of 100 to 300, is below the last variable point axis. In addition, a probability line for predicting sleep disorders ranging from 0.15 to 0.65 is at the bottom of the nomogram.

After the multivariate logistic regression analysis, the independent predictors such as diastolic blood pressure, respiratory rate, hemoglobin, delirium and cardiovascular disease (Table 2) were used for developing a nomogram. According to the odds ratio values of the predictors, a standard scoring system was established. And the score of each predictor to sleep disorders predictor was evaluated. Adding up scores of each predictor with a visualization score, the probability of predicting sleep disorders was effectively estimated. The total score of these predictors was 229, which corresponded to the sleep-disorder prediction probability of 0.522 (Figure 2).

### Validation of the Nomogram

**FIGURE 3:**
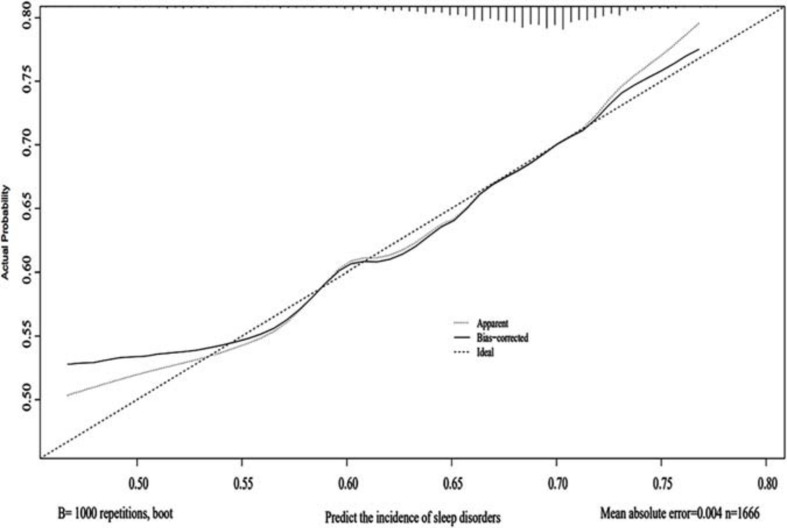
Calibration of the nomogram. The predictive performance for the incidence of sleep disorders was evaluated in the validation set, which was applied with the 1000 bootstrap resampling. The predicted probability and the observed probability are presented by the X and Y axes, respectively.

**FIGURE 4:**
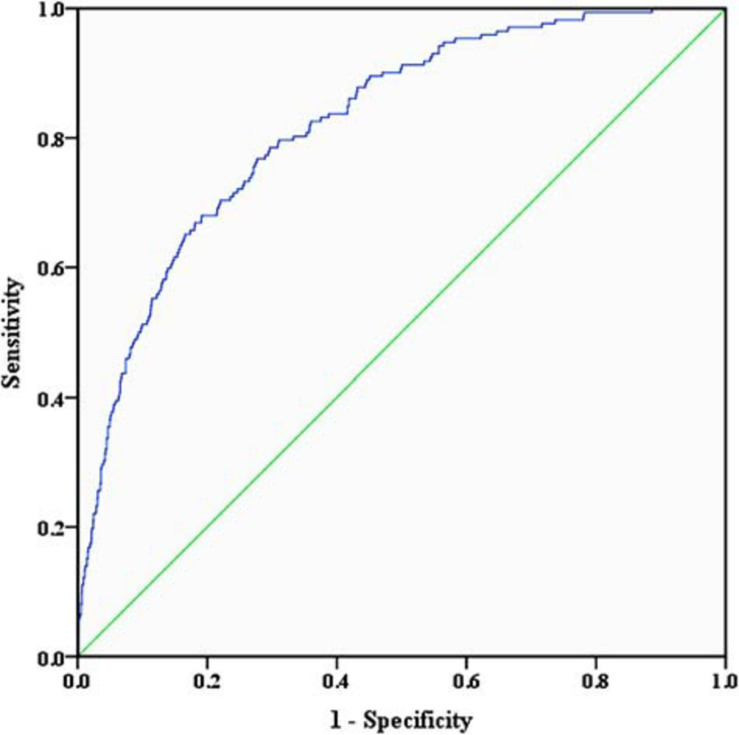
Receiver operating characteristic curve of the nomogram. The sensitivity and specificity of the ROC reflect the discrimination of the nomogram for predicting sleep disorders. The AUC of the nomogram was 0.822 (95%CI: 0.791–0.854) in the validation set. ROC, receiver operating characteristic curve; AUC, area under curve.

Using the 1000 bootstrap resampling method, the performance of the nomogram was internally validated in the validation set. The calibration plot of current depression rates suggested a high consistency between the observed and predicted values in the probability of sleep disorders (Figure 3). In terms of the accuracy of predicting sleep disorders, the sensitivity and specificity of the nomogram in the ROC were 75.6% and 72.9%, respectively. The AUC was 0.822 (95%CI: 0.791–0.854) (Figure 4).

### Clinical Utility of the Nomogram

**FIGURE 5:**
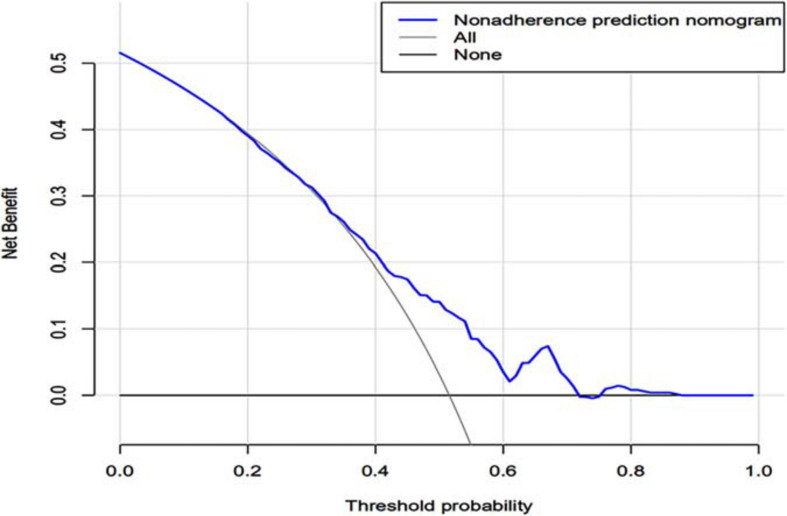
Decision curve analysis of the nomogram. X-axis and y-axis represent threshold probability and net benefit, respectively. For the clinical utility of the nomogram as a diagnostic model for sleep disorders, the net benefit curve is shown in the DCA. When the threshold value of the diagnostic model is between 0.50 and 0.99, the patients will obtain the corresponding net benefit as long as the therapeutic measure is taken. DCA, decision curve analysis.

In the DCA, the threshold probability of the nomogram ranged from 55% to 90%, which suggested a wide range of clinical utility. As long as the prediction probability of ICU patients with sleep disorders is between 55% and 90%, the nomogram will deliver the net benefit in varying degrees under the corresponding intervention measure. The clinical utility of the nomogram to predict sleep disorders is shown in Figure 5.

## Discussion

Based on the MIMIC-III database, we developed and validated the risk prediction model for non-specific sleep disorders in ICU patients. As far as we know, this is the first attempt to predict sleep disorders in the ICU. We successfully drew the visualization nomogram, which had a good performance in predicting sleep disorders. Moreover, the nomogram had sufficient net benefit for clinical application, in addition to good discrimination and calibration. In the clinical dataset, we found that the modifiable risk predictors made up of low diastolic blood pressure, low respiratory rate, low hemoglobin level, cardiovascular disease, and delirium were significantly bound up with sleep disorders in the ICU. Overall, our results show that the combined set of these seven predictors can be effectively used for the clinical prediction model of sleep disorders in ICU patients. Furthermore, a further prospective study will be necessary.

Intensive medicine is often accompanied by a high incidence and severity of sleep disorders in the ICU. According to the clinical practice guideline for sleep disruption in critically ill adults (Devlin et al., 2018), noise and light in the ICU environment, which were common interferences in the sleep-wake cycle, could be prevented and managed by non-pharmacological interventions (earplugs and eye masks), as well as melatonin. However, few high-level kinds of researches are known about the hidden risk factors in clinical datasets outside the environmental factors that not only have modifiable characteristics but also have predictive values. Most importantly, there is a lack of high-quality evidence to recommend the management of sleep disorders in the ICU. Also, the assessment and classification of sleep disorders are complicated (Auger et al., 2015; Smith et al., 2018), among which specific sleep disorders such as sleep-disordered breathing, central narcolepsy, and sleep-related movement disorder have little correlation with clinical characteristics in the ICU. Besides, specific sleep disorders lack predictive values due to specific causes and low incidences. In turn, non-specific sleep disorders were the main subtypes in ICU patients, which were the focus and difficulty of clinical prevention and management (Bani Younis and Hayajneh, 2018; Devlin et al., 2018). Therefore, except for the environmental factors, the other potential risk factors of non-specific sleep disorders should be well-considered in the management of ICU patients. For ICU patients, it is very important to accurately predict the modifiable risk factors prior to the occurrence of sleep disorders.

From previous studies, we have known some risk factors for sleep disorders in the ICU, such as vital signs, comorbid disease, medication and medical procedure (Pisani et al., 2015; Boyko et al., 2017; Devlin et al., 2018). However, there is no high consensus on the risk factors of sleep disorders in these studies. The two global public issues of hypertension and sleep disorders often coexist (Han et al., 2020), and sleep disorders are regarded as the risk factors of hypertension (Pepin et al., 2014; Lo et al., 2018). On the contrary, hypertension was not the risk factor of sleep disorders in our study. However, this does not mean that blood pressure has little effect on sleep. At least, high diastolic blood pressure was an independent risk factor for sleep disorders in our study. Another important vital sign, respiratory rate, also had the predictive value for sleep disorders according to our study. Although the underlying mechanism of these factors is unclear, their clinical predictive values are still great in the case of the comprehensive analysis of the other clinical features they accompany. As the state of critically ill patients is complicated, it is necessary to analyze the effects of multiple comorbidities on sleep. Our study showed that cardiovascular disease is an independent predictor for sleep disorders in comorbid diseases, and this result contributes to the interaction between cardiovascular disease and sleep disorders. Notably, delirium is also a common comorbid disease in critical illness. The occurring and worsening of delirium are independently associated with sleep disorders in the ICU (Devlin et al., 2018; Pisani and D’Ambrosio, 2020). In spite of a lot of literature regarding sleep on delirium (Flannery et al., 2016; Park and Lee, 2019), the link between sleep and delirium remains the significant concern in the ICU. With our study, we found that delirium was also an independent risk factor of sleep disorders, and this result provides a new window for peeping into the two-way interactions between sleep and delirium. This means that while preventing and managing delirium, sleep disorders can be reduced correspondingly.

Based on laboratory tests, it is also a focus of our study to seek potential biomarkers of sleep disorders. However, there is a lack of researches on potential risk predictors in laboratory tests for sleep disorders in the ICU. For this reason, our study analyzed conventional laboratory tests, including the levels of hemoglobin, platelets, potassium, PH, and glucose. Finally, it was confirmed that the low hemoglobin level was an independent risk predictor, so it can be used as a significant biomarker of sleep disorders. Despite some studies have shown that sickle cell anemia is closely associated with restless withdrawal syndrome and obstructive sleep apnea in specific sleep disorders (Rosen et al., 2014; Erdem et al., 2020), the association between anemia and non-specific sleep disorders is poorly understood. Our research explored this issue and confirmed that anemia is potentially linked to non-specific sleep disorders. Despite anemia interacts with sleep problems clinically (Chen-Edinboro et al., 2018; Liu et al., 2018), hemoglobin may be a higher predictive value. This is because that the diagnosis and classification of anemia are based on the threshold of hemoglobin decline, and hemoglobin itself level can be accurately and dynamically evaluated without considering the classification and etiology of anemia. In addition, hemoglobin has been proved to be a biomarker of sleep time and quality in the elderly (Jackowska et al., 2013). But most importantly, hemoglobin can also be recommended as an independent biomarker of sleep disorders in the ICU adult according to our study.

Due to the nature of the severe illness, ICU patients need a variety of medications and medical treatments, part of which may disturb sleep (Pisani et al., 2015). ICU patients need to use sedatives and analgesics to relieve discomfort and pain. Sedation does not mean natural sleep and damages sleep in turn. Benzodiazepines and propofol are common sedatives in the ICU. Long-term application of benzodiazepines can lead to an increase of shallow sleep (Weinhouse, 2008). And propofol not only reduces sleep quality but also greatly increases the risk of delirium (Kamdar et al., 2012; Telias and Wilcox, 2019). In addition, opioids of analgesics relieve severe pain, accompanied by inhibiting slow-wave sleep, increasing night awakening, and resulting in delirium (Al Mutair et al., 2020; Kamdar et al., 2012). However, our study did not show the adverse effects of the sedatives and analgesics on sleep. Despite dexmedetomidine showed the characteristic of a potential protective effect on sleep (Brito et al., 2020), this effect did not occur in our study. Therefore, it is necessary to find a balance between comfort and sleep. In addition, our study did not support the association of sleep disorders with antibiotics, cardiovascular drugs, and steroids, which is in contradiction with other studies (Kamdar et al., 2012; Pulak and Jensen, 2016). In our opinion, medications in the ICU may affect sleep, but the impact is limited. Many ICU patients need mechanical ventilation, but man-machine confrontation and gas exchange can disturb sleep and make sleep fragmentation (Hardin et al., 2006; Kamdar et al., 2012). Nevertheless, in the process of the mechanical ventilation, the application of sedatives and analgesics can eliminate their adverse effects on sleep in turn. Therefore, we should not take a negative attitude toward these medications and medical procedures, only because of their potential risks for sleep disorders. And we should consider the overall benefits of patients in the ICU, certainly including the normal sleep needs of patients.

## Limitations

There are some limitations to our study. First, due to the inherent limitations of the MIMIC-III database, we could not obtain the continuous, dynamic variables, and the diagnostic codes for sleep disorders were not the latest version. All data from the single database, not multicenter research, consequently our study could not confirm the causal relationship between these predictors and sleep disorders. Despite our study used propensity matching analysis to control the confounding factors of age and gender, other confounding factors were not controlled effectively due to the complexity of clinical data. Due to the exclusion of specific sleep disorders, the selective bias of our study was increased, and the extrapolation and general application value of this study were also reduced correspondingly. In particular, further prospective research is needed to evaluate its clinical value. In spite of these limitations, the main advantage of our study was that the data were based on a large population, covering the majority of ICU patients. Moreover, the high prediction of the model suggests strong internal validation of these results and significant clinical utility.

## Conclusion

In summary, our study presents a clinical prediction model for sleep disorders in ICU adults. For this population, giving priority to the risk predictors, especially the modifiable biomarkers such as hemoglobin, may be necessary. The model with high sensitivity and specificity may serve as a clinical tool for ICU adult individuals. Besides, ICU patients unaware of the risks will gain net benefit when applying the model. The model may be helpful for medical staff to take tailored interventions for clinical modifiable risks. It will contribute to the prevention and management of sleep disorders in ICU patients.

## Data Availability Statement

Publicly available datasets were analyzed in this study. This data can be found here: The MIMIC III database (version 1.4) is publically available from https://mimic.physionet.org/.

## Ethics Statement

Data was approved by the Massachusetts Institute of Technology (Cambridge, MA) and the Institutional Review Boards of Beth Israel Deaconess Medical Center (Boston, MA). Individual patient consent was not required, because the extraction of publicly available data for this study did not affect patient care and all data was anonymized.

## Author Contributions

HW and YL developed the framework of this study. ZY, JS, QZ, XZ, JG, and QW collected the data. YL and LZ analyzed the data. HW revised the manuscript and made the final version of the manuscript. All authors read and approved the final manuscript.

## Conflict of Interest

The authors declare that the research was conducted in the absence of any commercial or financial relationships that could be construed as a potential conflict of interest.

## Supplementary Material

Supplementary Material 1 Inclusion of patients with non-specific sleep disorders according to ICD-9 codes.

Supplementary Material 2 Exclusion of patients with specific sleep disorders according to ICD-9 codes.

Supplementary Material 3 Type of disease and ICD9-codes.

Supplementary Material 4 Patients with cognitive impairment from the MIMIC-III database according to ICD9-codes.

Supplementary Material 5 Data Profiling Report.

## Abbreviations

AUC: area under curve
CI: confidence Interval
DCA: decision curve analysis
GCS: Glasgow coma scale
ICD-9, International Classification of Diseases: Ninth Revision
ICU: intensive care unit
MIMIC-III: Medical Information Mart for Intensive Care III
OR: odds ratio
ROC: receiver operating characteristic curve
SAPS II: simplified acute physiology score II
SOFA: sequential organ failure assessment
