# Supplementary material for: Development and Validation of a Clinical Prediction Model for Sleep Disorders in the ICU: A Retrospective Cohort Study
Source: Front Neurosci. 2021 Apr 16;15:644845. doi: 10.3389/fnins.2021.644845 (PMC8085546; doi:10.3389/fnins.2021.644845)
Supplement: Supplementary Material 1 — Inclusion of patients with non-specific sleep disorders according to ICD-9 codes. [file Table_1.docx]

| **Supplementary material 1** Inclusion of patients with non-specific sleep disorders according to ICD-9 codes | | |
| --- | --- | --- |
| **Disease** | ICD9-Code | Description |
| Sleep disorders |  |  |
|  | 30741 | Transient disorder of initiating or maintaining sleep |
|  | 30742 | Persistent disorder of initiating or maintaining sleep |
|  | 32700 | Organic insomnia, unspecified |
|  | 78050 | Sleep disturbance, unspecified |
|  | 78051 | Insomnia with sleep apnea, unspecified |
|  | 78052 | Insomnia, unspecified |
|  | V694 | Lack of adequate sleep |
|  | 32720 | Organic sleep apnea, unspecified |
|  | 78057 | Unspecified sleep apnea |
|  | 30743 | Transient disorder of initiating or maintaining wakefulness |
|  | 30744 | Persistent disorder of initiating or maintaining wakefulness |
|  | 32710 | Organic hypersomnia, unspecified |
|  | 78053 | Hypersomnia with sleep apnea, unspecified |
|  | 78054 | Hypersomnia, unspecified |
|  | 78055 | Disruption of 24 hour sleep wake cycle, unspecified |
|  | 30745 | Circadian rhythm sleep disorder of nonorganic origin |
|  | 32730 | Circadian rhythm sleep disorder, unspecified |
|  | 32731 | Circadian rhythm sleep disorder, delayed sleep phase type |
|  | 32732 | Circadian rhythm sleep disorder, advanced sleep phase type |
|  | 32733 | Circadian rhythm sleep disorder, irregular sleep-wake type |
|  | 32734 | Circadian rhythm sleep disorder, free-running type |
|  | 32735 | Circadian rhythm sleep disorder, jet lag type |
|  | 32736 | Circadian rhythm sleep disorder, shift work type |
|  | 32737 | Circadian rhythm sleep disorder in conditions classified elsewhere |
|  | 32739 | Other circadian rhythm sleep disorder |
|  | 32740 | Organic parasomnia, unspecified |
|  | 32741 | Confusional arousals |
|  | 32742 | REM sleep behavior disorder |
|  | 32743 | Recurrent isolated sleep paralysis |
|  | 32744 | Parasomnia in conditions classified elsewhere |
|  | 30746 | Sleep arousal disorder |
|  | 30747 | Other dysfunctions of sleep stages or arousal from sleep |
|  | 78056 | Dysfunctions associated with sleep stages or arousal from sleep |
|  | 78059 | Other sleep disturbances |
|  | 30740 | Nonorganic sleep disorder, unspecified |
|  | 30748 | Repetitive intrusions of sleep |
| ICD9:Inter national Classification of Diseases, Ninth Revision | | |
