# Supplementary material for: Development and Validation of a Clinical Prediction Model for Sleep Disorders in the ICU: A Retrospective Cohort Study
Source: Front Neurosci. 2021 Apr 16;15:644845. doi: 10.3389/fnins.2021.644845 (PMC8085546; doi:10.3389/fnins.2021.644845)
Supplement: Supplementary Material 2 — Exclusion of patients with specific sleep disorders according to ICD-9 codes. [file Table_2.docx]

| **Supplementary material 2**  Exclusion of patients with specific sleep disorders according to ICD-9 codes | | |
| --- | --- | --- |
| Disease | ICD9-Codes |  |
| Sleep disorders |  |  |
|  | 4672 | Fatal familial insomnia |
|  | 32701 | Insomnia due to medical condition classified elsewhere |
|  | 32702 | Insomnia due to mental disorder |
|  | V695 | Behavioral insomnia of childhood |
|  | 32709 | Other organic insomnia |
|  | 32721 | Primary central sleep apnea |
|  | 32722 | High altitude periodic breathing |
|  | 32723 | Obstructive sleep apnea (adult)(pediatric) |
|  | 32724 | Idiopathic sleep related non-obstructive alveolar hypoventilation |
|  | 32725 | Congenital central alveolar hypoventilation syndrome |
|  | 32726 | Sleep related hypoventilation/hypoxemia in conditions classifiable elsewhere |
|  | 32727 | Central sleep apnea in conditions classified elsewhere |
|  | 32729 | Other organic sleep apnea |
|  | 32711 | Idiopathic hypersomnia with long sleep time |
|  | 32712 | Idiopathic hypersomnia without long sleep time |
|  | 32713 | Recurrent hypersomnia |
|  | 32714 | Hypersomnia due to medical condition classified elsewhere |
|  | 32715 | Hypersomnia due to mental disorder |
|  | 32719 | Other organic hypersomnia |
|  | 34710 | Narcolepsy in conditions classified elsewhere, without cataplexy |
|  | 34711 | Narcolepsy in conditions classified elsewhere, with cataplexy |
|  | 34700 | Narcolepsy, without cataplexy |
|  | 34701 | Narcolepsy, with cataplexy |
|  | 32749 | Other organic parasomnia |
|  | 30749 | Other specific disorders of sleep of nonorganic origin |
|  | 29182 | Alcohol induced sleep disorders |
|  | 32751 | Periodic limb movement disorder |
|  | 32752 | Sleep related leg cramps |
|  | 32753 | Sleep related bruxism |
|  | 32759 | Other organic sleep related movement disorders |
|  | 3278 | Other organic sleep disorders |
|  | 33394 | Restless legs syndrome (RLS) |
|  | 78058 | Sleep related movement disorder, unspecified |
| ICD9:Inter national Classification of Diseases, Ninth Revision | | |
