# Supplementary material for: Development and Validation of a Clinical Prediction Model for Sleep Disorders in the ICU: A Retrospective Cohort Study
Source: Front Neurosci. 2021 Apr 16;15:644845. doi: 10.3389/fnins.2021.644845 (PMC8085546; doi:10.3389/fnins.2021.644845)
Supplement: Supplementary Material 3 — Type of disease and ICD9-codes. [file Table_3.docx]

| **Supplementary material 3** Type of disease and ICD9-codes | | |  |  |  |  |  |  |  |  |  |
| --- | --- | --- | --- | --- | --- | --- | --- | --- | --- | --- | --- |
| Disease | ICD9-Code | Description |  |  |  |  |  |  |  |  |  |
| Hypertension |  |  |  |  |  |  |  |  |  |  |  |
|  | 64222 | Other pre-existing hypertension, complicating pregnancy, childbirth,  and the puerperium, delivered, with mention of postpartum complication |  |  |  |  |  |  |  |  |  |
|  | 64200 | Benign essential hypertension complicating pregnancy, childbirth,  and the puerperium, unspecified as to episode of care or not applicable |  |  |  |  |  |  |  |  |  |
|  | 64201 | Benign essential hypertension complicating pregnancy, childbirth,  and the puerperium, delivered, with or without mention of antepartum condition |  |  |  |  |  |  |  |  |  |
|  | 64202 | Benign essential hypertension, complicating pregnancy, childbirth,  and the puerperium, delivered, with mention of postpartum complication |  |  |  |  |  |  |  |  |  |
|  | 64203 | Benign essential hypertension complicating pregnancy, childbirth,  and the puerperium, antepartum condition or complication |  |  |  |  |  |  |  |  |  |
|  | 64204 | Benign essential hypertension complicating pregnancy, childbirth,  and the puerperium, postpartum condition or complication |  |  |  |  |  |  |  |  |  |
|  | 64210 | Hypertension secondary to renal disease, complicating pregnancy, childbirth,  and the puerperium, unspecified as to episode of care or not applicable |  |  |  |  |  |  |  |  |  |
|  | 64211 | Hypertension secondary to renal disease, complicating pregnancy, childbirth,  and the puerperium, delivered, with or without mention of antepartum condition |  |  |  |  |  |  |  |  |  |
|  | 64212 | Hypertension secondary to renal disease, complicating pregnancy, childbirth,  and the puerperium, delivered, with mention of postpartum complication |  |  |  |  |  |  |  |  |  |
|  | 64213 | Hypertension secondary to renal disease, complicating pregnancy, childbirth,  and the puerperium, antepartum condition or complication |  |  |  |  |  |  |  |  |  |
|  | 64214 | Hypertension secondary to renal disease, complicating pregnancy, childbirth,  and the puerperium, postpartum condition or complication |  |  |  |  |  |  |  |  |  |
|  | 64220 | Other pre-existing hypertension complicating pregnancy, childbirth,  and the puerperium, unspecified as to episode of care or not applicable |  |  |  |  |  |  |  |  |  |
|  | 64221 | Other pre-existing hypertension, complicating pregnancy, childbirth,  and the puerperium, delivered, with or without mention of antepartum condition |  |  |  |  |  |  |  |  |  |
|  | 64223 | Other pre-existing hypertension, complicating pregnancy, childbirth,  and the puerperium, antepartum condition or complication |  |  |  |  |  |  |  |  |  |
|  | 64224 | Other pre-existing hypertension,complicating pregnancy, childbirth,  and the puerperium, , postpartum condition or complication |  |  |  |  |  |  |  |  |  |
|  | 64270 | Pre-eclampsia or eclampsia superimposed on pre-existing hypertension,  unspecified as to episode of care or not applicable |  |  |  |  |  |  |  |  |  |
|  | 64271 | Pre-eclampsia or eclampsia superimposed on pre-existing hypertension,  delivered, with or without mention of antepartum condition |  |  |  |  |  |  |  |  |  |
|  | 64272 | Pre-eclampsia or eclampsia superimposed on pre-existing hypertension,  delivered, with mention of postpartum complication |  |  |  |  |  |  |  |  |  |
|  | 64273 | Pre-eclampsia or eclampsia superimposed on pre-existing hypertension,  antepartum condition or complication |  |  |  |  |  |  |  |  |  |
|  | 64274 | Pre-eclampsia or eclampsia superimposed on pre-existing hypertension,  postpartum condition or complication |  |  |  |  |  |  |  |  |  |
|  | 64290 | Unspecified hypertension complicating pregnancy, childbirth, or the puerperium,  unspecified as to episode of care or not applicable |  |  |  |  |  |  |  |  |  |
|  | 7600 | Maternal hypertensive disorders affecting fetus or newborn |  |  |  |  |  |  |  |  |  |
|  | 9726 | Poisoning by other antihypertensive agents |  |  |  |  |  |  |  |  |  |
|  | 99791 | Complications affecting other specified body systems, not elsewhere classified, hypertension |  |  |  |  |  |  |  |  |  |
|  | E9426 | Other antihypertensive agents causing adverse effects in therapeutic use |  |  |  |  |  |  |  |  |  |
|  | 36042 | Blind hypertensive eye |  |  |  |  |  |  |  |  |  |
|  | 36211 | Hypertensive retinopathy |  |  |  |  |  |  |  |  |  |
|  | 4010 | Malignant essential hypertension |  |  |  |  |  |  |  |  |  |
|  | 4011 | Benign essential hypertension |  |  |  |  |  |  |  |  |  |
|  | 4019 | Unspecified essential hypertension |  |  |  |  |  |  |  |  |  |
|  | 40200 | Malignant hypertensive heart disease without heart failure |  |  |  |  |  |  |  |  |  |
|  | 40201 | Malignant hypertensive heart disease with heart failure |  |  |  |  |  |  |  |  |  |
|  | 40210 | Benign hypertensive heart disease without heart failure |  |  |  |  |  |  |  |  |  |
|  | 40211 | Benign hypertensive heart disease with heart failure |  |  |  |  |  |  |  |  |  |
|  | 40290 | Unspecified hypertensive heart disease without heart failure |  |  |  |  |  |  |  |  |  |
|  | 40291 | Unspecified hypertensive heart disease with heart failure |  |  |  |  |  |  |  |  |  |
|  | 40300 | Hypertensive chronic kidney disease, malignant,  with chronic kidney disease stage I through stage IV, or unspecified |  |  |  |  |  |  |  |  |  |
|  | 40301 | Hypertensive chronic kidney disease, malignant,  with chronic kidney disease stage V or end stage renal disease |  |  |  |  |  |  |  |  |  |
|  | 40310 | Hypertensive chronic kidney disease, benign,  with chronic kidney disease stage I through stage IV, or unspecified |  |  |  |  |  |  |  |  |  |
|  | 40311 | Hypertensive chronic kidney disease, benign,  with chronic kidney disease stage V or end stage renal disease |  |  |  |  |  |  |  |  |  |
|  | 40390 | Hypertensive chronic kidney disease, unspecified,  with chronic kidney disease stage I through stage IV, or unspecified |  |  |  |  |  |  |  |  |  |
|  | 40391 | Hypertensive chronic kidney disease, unspecified,  with chronic kidney disease stage V or end stage renal disease |  |  |  |  |  |  |  |  |  |
|  | 40400 | Hypertensive heart and chronic kidney disease, malignant,  without heart failure and with chronic kidney disease stage I through stage IV,  or unspecified |  |  |  |  |  |  |  |  |  |
|  | 40401 | Hypertensive heart and chronic kidney disease, malignant,  with heart failure and with chronic kidney disease stage I through stage IV, or unspecified |  |  |  |  |  |  |  |  |  |
|  | 40402 | Hypertensive heart and chronic kidney disease, malignant,  without heart failure and with chronic kidney disease stage V or end stage renal disease |  |  |  |  |  |  |  |  |  |
|  | 40403 | Hypertensive heart and chronic kidney disease, malignant,  with heart failure and with chronic kidney disease stage V or end stage renal disease |  |  |  |  |  |  |  |  |  |
|  | 40410 | Hypertensive heart and chronic kidney disease, benign,  without heart failure and with chronic kidney disease stage I through stage IV, or unspecified |  |  |  |  |  |  |  |  |  |
|  | 40411 | Hypertensive heart and chronic kidney disease, benign,  with heart failure and with chronic kidney disease stage I through stage IV, or unspecified |  |  |  |  |  |  |  |  |  |
|  | 40412 | Hypertensive heart and chronic kidney disease, benign,  without heart failure and with chronic kidney disease stage V or end stage renal disease |  |  |  |  |  |  |  |  |  |
|  | 40413 | Hypertensive heart and chronic kidney disease, benign,  with heart failure and chronic kidney disease stage V or end stage renal disease |  |  |  |  |  |  |  |  |  |
|  | 40490 | Hypertensive heart and chronic kidney disease, unspecified,  without heart failure and with chronic kidney disease stage I through stage IV, or unspecified |  |  |  |  |  |  |  |  |  |
|  | 40491 | Hypertensive heart and chronic kidney disease, unspecified,  with heart failure and with chronic kidney disease stage I through stage IV, or unspecified |  |  |  |  |  |  |  |  |  |
|  | 40492 | Hypertensive heart and chronic kidney disease, unspecified,  without heart failure and with chronic kidney disease stage V or end stage renal disease |  |  |  |  |  |  |  |  |  |
|  | 40493 | Hypertensive heart and chronic kidney disease, unspecified,  with heart failure and chronic kidney disease stage V or end stage renal disease |  |  |  |  |  |  |  |  |  |
|  | 40501 | Malignant renovascular hypertension |  |  |  |  |  |  |  |  |  |
|  | 40509 | Other malignant secondary hypertension |  |  |  |  |  |  |  |  |  |
|  | 40511 | Benign renovascular hypertension |  |  |  |  |  |  |  |  |  |
|  | 40519 | Other benign secondary hypertension |  |  |  |  |  |  |  |  |  |
|  | 40599 | Other unspecified secondary hypertension |  |  |  |  |  |  |  |  |  |
|  | 40591 | Unspecified renovascular hypertension |  |  |  |  |  |  |  |  |  |
|  | 45930 | Chronic venous hypertension without complications |  |  |  |  |  |  |  |  |  |
|  | 45931 | Chronic venous hypertension with ulcer |  |  |  |  |  |  |  |  |  |
|  | 45932 | Chronic venous hypertension with inflammation |  |  |  |  |  |  |  |  |  |
|  | 45933 | Chronic venous hypertension with ulcer and inflammation |  |  |  |  |  |  |  |  |  |
|  | 45939 | Chronic venous hypertension with other complication |  |  |  |  |  |  |  |  |  |
| Diabetes |  |  |  |  |  |  |  |  |  |  |  |
|  | E9323 | Insulins and antidiabetic agents causing adverse effects in therapeutic use |  |  |  |  |  |  |  |  |  |
|  | 9623 | Poisoning by insulins and antidiabetic agents |  |  |  |  |  |  |  |  |  |
|  | 25000 | Diabetes mellitus without mention of complication,  type II or unspecified type, not stated as uncontrolled |  |  |  |  |  |  |  |  |  |
|  | 25001 | Diabetes mellitus without mention of complication, t  ype I [juvenile type], not stated as uncontrolled |  |  |  |  |  |  |  |  |  |
|  | 25002 | Diabetes mellitus without mention of complication,  type II or unspecified type, uncontrolled |  |  |  |  |  |  |  |  |  |
|  | 25003 | Diabetes mellitus without mention of complication, type I [juvenile type], uncontrolled |  |  |  |  |  |  |  |  |  |
|  | 25010 | Diabetes with ketoacidosis, type II or unspecified type, not stated as uncontrolled |  |  |  |  |  |  |  |  |  |
|  | 25011 | Diabetes with ketoacidosis, type I [juvenile type], not stated as uncontrolled |  |  |  |  |  |  |  |  |  |
|  | 25012 | Diabetes with ketoacidosis, type II or unspecified type, uncontrolled |  |  |  |  |  |  |  |  |  |
|  | 25013 | Diabetes with ketoacidosis, type I [juvenile type], uncontrolled |  |  |  |  |  |  |  |  |  |
|  | 25020 | Diabetes with hyperosmolarity, type II or unspecified type, not stated as uncontrolled |  |  |  |  |  |  |  |  |  |
|  | 25021 | Diabetes with hyperosmolarity, type I [juvenile type], not stated as uncontrolled |  |  |  |  |  |  |  |  |  |
|  | 25022 | Diabetes with hyperosmolarity, type II or unspecified type, uncontrolled |  |  |  |  |  |  |  |  |  |
|  | 25023 | Diabetes with hyperosmolarity, type I [juvenile type], uncontrolled |  |  |  |  |  |  |  |  |  |
|  | 25030 | Diabetes with other coma, type II or unspecified type, not stated as uncontrolled |  |  |  |  |  |  |  |  |  |
|  | 25031 | Diabetes with other coma, type I [juvenile type], not stated as uncontrolled |  |  |  |  |  |  |  |  |  |
|  | 25032 | Diabetes with other coma, type II or unspecified type, uncontrolled |  |  |  |  |  |  |  |  |  |
|  | 25033 | Diabetes with other coma, type I [juvenile type], uncontrolled |  |  |  |  |  |  |  |  |  |
|  | 25040 | Diabetes with renal manifestations, type II or unspecified type, not stated as uncontrolled |  |  |  |  |  |  |  |  |  |
|  | 24900 | Secondary diabetes mellitus without mention of complication,  not stated as uncontrolled, or unspecified |  |  |  |  |  |  |  |  |  |
|  | 24901 | Secondary diabetes mellitus without mention of complication, uncontrolled |  |  |  |  |  |  |  |  |  |
|  | 24910 | Secondary diabetes mellitus with ketoacidosis, not stated as uncontrolled, or unspecified |  |  |  |  |  |  |  |  |  |
|  | 24911 | Secondary diabetes mellitus with ketoacidosis, uncontrolled |  |  |  |  |  |  |  |  |  |
|  | 24920 | Secondary diabetes mellitus with hyperosmolarity, not stated as uncontrolled, or unspecified |  |  |  |  |  |  |  |  |  |
|  | 24921 | Secondary diabetes mellitus with hyperosmolarity, uncontrolled |  |  |  |  |  |  |  |  |  |
|  | 24930 | Secondary diabetes mellitus with other coma, not stated as uncontrolled, or unspecified |  |  |  |  |  |  |  |  |  |
|  | 24931 | Secondary diabetes mellitus with other coma, uncontrolled |  |  |  |  |  |  |  |  |  |
|  | 24940 | Secondary diabetes mellitus with renal manifestations,  not stated as uncontrolled, or unspecified |  |  |  |  |  |  |  |  |  |
|  | 24941 | Secondary diabetes mellitus with renal manifestations, uncontrolled |  |  |  |  |  |  |  |  |  |
|  | 24950 | Secondary diabetes mellitus with ophthalmic manifestations,  not stated as uncontrolled, or unspecified |  |  |  |  |  |  |  |  |  |
|  | 24951 | Secondary diabetes mellitus with ophthalmic manifestations, uncontrolled |  |  |  |  |  |  |  |  |  |
|  | 24960 | Secondary diabetes mellitus with neurological manifestations,  not stated as uncontrolled, or unspecified |  |  |  |  |  |  |  |  |  |
|  | 24961 | Secondary diabetes mellitus with neurological manifestations, uncontrolled |  |  |  |  |  |  |  |  |  |
|  | 24970 | Secondary diabetes mellitus with peripheral circulatory disorders,  not stated as uncontrolled, or unspecified |  |  |  |  |  |  |  |  |  |
|  | 24971 | Secondary diabetes mellitus with peripheral circulatory disorders, uncontrolled |  |  |  |  |  |  |  |  |  |
|  | 24980 | Secondary diabetes mellitus with other specified manifestations,  not stated as uncontrolled, or unspecified |  |  |  |  |  |  |  |  |  |
|  | 24981 | Secondary diabetes mellitus with other specified manifestations, uncontrolled |  |  |  |  |  |  |  |  |  |
|  | 24990 | Secondary diabetes mellitus with unspecified complication,  not stated as uncontrolled, or unspecified |  |  |  |  |  |  |  |  |  |
|  | 24991 | Secondary diabetes mellitus with unspecified complication, uncontrolled |  |  |  |  |  |  |  |  |  |
|  | 25041 | Diabetes with renal manifestations, type I [juvenile type], not stated as uncontrolled |  |  |  |  |  |  |  |  |  |
|  | 25042 | Diabetes with renal manifestations, type II or unspecified type, uncontrolled |  |  |  |  |  |  |  |  |  |
|  | 25043 | Diabetes with renal manifestations, type I [juvenile type], uncontrolled |  |  |  |  |  |  |  |  |  |
|  | 25050 | Diabetes with ophthalmic manifestations, type II or unspecified type,  not stated as uncontrolled |  |  |  |  |  |  |  |  |  |
|  | 25051 | Diabetes with ophthalmic manifestations, type I [juvenile type], not stated as uncontrolled |  |  |  |  |  |  |  |  |  |
|  | 25052 | Diabetes with ophthalmic manifestations, type II or unspecified type, uncontrolled |  |  |  |  |  |  |  |  |  |
|  | 25053 | Diabetes with ophthalmic manifestations, type I [juvenile type], uncontrolled |  |  |  |  |  |  |  |  |  |
|  | 25060 | Diabetes with neurological manifestations, type II or unspecified type,  not stated as uncontrolled |  |  |  |  |  |  |  |  |  |
|  | 25061 | Diabetes with neurological manifestations, type I [juvenile type], not stated as uncontrolled |  |  |  |  |  |  |  |  |  |
|  | 25062 | Diabetes with neurological manifestations, type II or unspecified type, uncontrolled |  |  |  |  |  |  |  |  |  |
|  | 25063 | Diabetes with neurological manifestations, type I [juvenile type], uncontrolled |  |  |  |  |  |  |  |  |  |
|  | 25070 | Diabetes with peripheral circulatory disorders, type II or unspecified type,  not stated as uncontrolled |  |  |  |  |  |  |  |  |  |
|  | 25071 | Diabetes with peripheral circulatory disorders, type I [juvenile type], not stated as uncontrolled |  |  |  |  |  |  |  |  |  |
|  | 25072 | Diabetes with peripheral circulatory disorders, type II or unspecified type, uncontrolled |  |  |  |  |  |  |  |  |  |
|  | 25073 | Diabetes with peripheral circulatory disorders, type I [juvenile type], uncontrolled |  |  |  |  |  |  |  |  |  |
|  | 25080 | Diabetes with other specified manifestations, type II or unspecified type, not stated as uncontrolled |  |  |  |  |  |  |  |  |  |
|  | 25081 | Diabetes with other specified manifestations, type I [juvenile type], not stated as uncontrolled |  |  |  |  |  |  |  |  |  |
|  | 25082 | Diabetes with other specified manifestations, type II or unspecified type, uncontrolled |  |  |  |  |  |  |  |  |  |
|  | 25083 | Diabetes with other specified manifestations, type I [juvenile type], uncontrolled |  |  |  |  |  |  |  |  |  |
|  | 25090 | Diabetes with unspecified complication, type II or unspecified type, not stated as uncontrolled |  |  |  |  |  |  |  |  |  |
|  | 25091 | Diabetes with unspecified complication, type I [juvenile type], not stated as uncontrolled |  |  |  |  |  |  |  |  |  |
|  | 25092 | Diabetes with unspecified complication, type II or unspecified type, uncontrolled |  |  |  |  |  |  |  |  |  |
|  | 25093 | Diabetes with unspecified complication, type I [juvenile type], uncontrolled |  |  |  |  |  |  |  |  |  |
| Chronic Liver disease |  |  |  |  |  |  |  |  |  |  |  |
|  | 0701 | Viral hepatitis A without mention of hepatic coma |  |  |  |  |  | 0701 |  | Viral hepatitis A without mention of hepatic coma |  |
|  | 07020 | Viral hepatitis B with hepatic coma, acute or unspecified, without mention of hepatitis delta |  |  |  |  |  | 07020 |  | Viral hepatitis B with hepatic coma, acute or unspecified, without mention of hepatitis delta |  |
|  | 07030 | Viral hepatitis B without mention of hepatic coma, acute or unspecified,  without mention of hepatitis |  |  |  |  |  | 07030 |  | Viral hepatitis B without mention of hepatic coma, acute or unspecified, without mention of hepatitis |  |
|  | 07031 | Viral hepatitis B without mention of hepatic coma, acute or unspecified, with hepatitis delta |  |  |  |  |  | 07031 |  | Viral hepatitis B without mention of hepatic coma, acute or unspecified, with hepatitis delta |  |
|  | 07032 | Chronic viral hepatitis B without mention of hepatic coma without mention of hepatitis delta |  |  |  |  |  | 07032 |  | Chronic viral hepatitis B without mention of hepatic coma without mention of hepatitis delta |  |
|  | 07033 | Chronic viral hepatitis B without mention of hepatic coma with hepatitis delta |  |  |  |  |  | 07033 |  | Chronic viral hepatitis B without mention of hepatic coma with hepatitis delta |  |
|  | 07051 | Acute hepatitis C without mention of hepatic coma |  |  |  |  |  | 07051 |  | Acute hepatitis C without mention of hepatic coma |  |
|  | 07052 | Hepatitis delta without mention of active hepatitis B disease or hepatic coma |  |  |  |  |  | 07052 |  | Hepatitis delta without mention of active hepatitis B disease or hepatic coma |  |
|  | 07053 | Hepatitis E without mention of hepatic coma |  |  |  |  |  | 07053 |  | Hepatitis E without mention of hepatic coma |  |
|  | 07054 | Chronic hepatitis C without mention of hepatic coma |  |  |  |  |  | 07054 |  | Chronic hepatitis C without mention of hepatic coma |  |
|  | 07070 | Unspecified viral hepatitis C without hepatic coma |  |  |  |  |  | 07070 |  | Unspecified viral hepatitis C without hepatic coma |  |
|  | 0709 | Unspecified viral hepatitis without mention of hepatic coma |  |  |  |  |  | 0709 |  | Unspecified viral hepatitis without mention of hepatic coma |  |
|  | 5712 | Alcoholic cirrhosis of liver |  |  |  |  |  | 5712 |  | Alcoholic cirrhosis of liver |  |
|  | 5713 | Alcoholic liver damage, unspecified |  |  |  |  |  | 5713 |  | Alcoholic liver damage, unspecified |  |
|  | 57140 | Chronic hepatitis, unspecified |  |  |  |  |  | 57140 |  | Chronic hepatitis, unspecified |  |
|  | 57141 | Chronic persistent hepatitis |  |  |  |  |  | 57141 |  | Chronic persistent hepatitis |  |
|  | 57142 | Autoimmune hepatitis |  |  |  |  |  | 57142 |  | Autoimmune hepatitis |  |
|  | 57149 | Other chronic hepatitis |  |  |  |  |  | 57149 |  | Other chronic hepatitis |  |
|  | 5715 | Cirrhosis of liver without mention of alcohol |  |  |  |  |  | 5715 |  | Cirrhosis of liver without mention of alcohol |  |
|  | 5716 | Biliary cirrhosis |  |  |  |  |  | 5716 |  | Biliary cirrhosis |  |
|  | 5718 | Other chronic nonalcoholic liver disease |  |  |  |  |  | 5718 |  | Other chronic nonalcoholic liver disease |  |
|  | 5719 | Unspecified chronic liver disease without mention of alcohol |  |  |  |  |  | 5719 |  | Unspecified chronic liver disease without mention of alcohol |  |
|  | 5724 | Hepatorenal syndrome |  |  |  |  |  | 5724 |  | Hepatorenal syndrome |  |
|  | 5728 | Other sequelae of chronic liver disease |  |  |  |  |  | 5728 |  | Other sequelae of chronic liver disease |  |
|  | 5738 | Other specified disorders of liver |  |  |  |  |  | 5738 |  | Other specified disorders of liver |  |
|  | 5735 | Hepatopulmonary syndrome |  |  |  |  |  | 5735 |  | Hepatopulmonary syndrome |  |
|  | 5734 | Hepatic infarction |  |  |  |  |  | 5734 |  | Hepatic infarction |  |
|  | 5733 | Hepatitis, unspecified |  |  |  |  |  | 5733 |  | Hepatitis, unspecified |  |
|  | 5732 | Hepatitis in other infectious diseases classified elsewhere |  |  |  |  |  | 5732 |  | Hepatitis in other infectious diseases classified elsewhere |  |
|  | 5731 | Hepatitis in viral diseases classified elsewhere |  |  |  |  |  | 5731 |  | Hepatitis in viral diseases classified elsewhere |  |
|  | 5730 | Chronic passive congestion of liver |  |  |  |  |  | 5730 |  | Chronic passive congestion of liver |  |
|  | V0260 | Viral hepatitis carrier, unspecified |  |  |  |  |  | V0260 |  | Viral hepatitis carrier, unspecified |  |
|  | V0261 | Hepatitis B | carrier |  |  |  |  | V0261 |  | Hepatitis B | carrier |
|  | V0262 | Hepatitis C | carrier |  |  |  |  | V0262 |  | Hepatitis C | carrier |
|  | V0269 | Other viral hepatitis carrier |  |  |  |  |  | V0269 |  | Other viral hepatitis carrier |  |
| Cardiovascular diseases |  |  |  |  |  |  |  |  |  |  |  |
|  |  |  |  |  |  |  |  |  |  |  |  |
|  | 9381 | Syphilitic pericarditis |  |  |  |  |  |  |  |  |  |
|  | 9382 | Syphilitic myocarditis |  |  |  |  |  |  |  |  |  |
|  | 9883 | Gonococcal pericarditis |  |  |  |  |  |  |  |  |  |
|  | 9884 | Gonococcal endocarditis |  |  |  |  |  |  |  |  |  |
|  | 9885 | Other gonococcal heart disease |  |  |  |  |  |  |  |  |  |
|  | 11513 | Infection by Histoplasma duboisii, pericarditis |  |  |  |  |  |  |  |  |  |
|  | 11514 | Infection by Histoplasma duboisii, endocarditis |  |  |  |  |  |  |  |  |  |
|  | 39890 | Rheumatic heart disease, unspecified |  |  |  |  |  |  |  |  |  |
|  | 39899 | Other rheumatic heart diseases |  |  |  |  |  |  |  |  |  |
|  | 40201 | Malignant hypertensive heart disease with heart failure |  |  |  |  |  |  |  |  |  |
|  | 40211 | Benign hypertensive heart disease with heart failure |  |  |  |  |  |  |  |  |  |
|  | 40290 | Unspecified hypertensive heart disease without heart failure |  |  |  |  |  |  |  |  |  |
|  | 40291 | Unspecified hypertensive heart disease with heart failure |  |  |  |  |  |  |  |  |  |
|  | 40401 | Hypertensive heart and chronic kidney disease, malignant,  with heart failure and with chronic kidney disease stage I through stage IV, or unspecified |  |  |  |  |  |  |  |  |  |
|  | 40403 | Hypertensive heart and chronic kidney disease, malignant,  with heart failure and with chronic kidney disease stage V or end stage renal disease |  |  |  |  |  |  |  |  |  |
|  | 40411 | Hypertensive heart and chronic kidney disease, benign,  with heart failure and with chronic kidney disease stage I through stage IV, or unspecified |  |  |  |  |  |  |  |  |  |
|  | 40413 | Hypertensive heart and chronic kidney disease, benign,  with heart failure and chronic kidney disease stage V or end stage renal disease |  |  |  |  |  |  |  |  |  |
|  | 40491 | Hypertensive heart and chronic kidney disease, unspecified,  with heart failure and with chronic kidney disease stage I through stage IV, or unspecified |  |  |  |  |  |  |  |  |  |
|  | 40493 | Hypertensive heart and chronic kidney disease, unspecified,  with heart failure and chronic kidney disease stage V or end stage renal disease |  |  |  |  |  |  |  |  |  |
|  | 41000 | Acute myocardial infarction of anterolateral wall, episode of care |  |  |  |  |  |  |  |  |  |
|  | 41001 | Acute myocardial infarction of anterolateral wall, initial episode of care |  |  |  |  |  |  |  |  |  |
|  | 41002 | Acute myocardial infarction of anterolateral wall, subsequent episode of care |  |  |  |  |  |  |  |  |  |
|  | 41010 | Acute myocardial infarction of other anterior wall, episode of care unspecified |  |  |  |  |  |  |  |  |  |
|  | 41011 | Acute myocardial infarction of other anterior wall, initial episode of care |  |  |  |  |  |  |  |  |  |
|  | 41012 | Acute myocardial infarction of other anterior wall, subsequent episode of care |  |  |  |  |  |  |  |  |  |
|  | 41020 | Acute myocardial infarction of inferolateral wall, episode of care unspecified |  |  |  |  |  |  |  |  |  |
|  | 41021 | Acute myocardial infarction of inferolateral wall, initial episode of care |  |  |  |  |  |  |  |  |  |
|  | 41022 | Acute myocardial infarction of inferolateral wall, subsequent episode of care |  |  |  |  |  |  |  |  |  |
|  | 41030 | Acute myocardial infarction of inferoposterior wall, episode of care unspecified |  |  |  |  |  |  |  |  |  |
|  | 41031 | Acute myocardial infarction of inferoposterior wall, initial episode of care |  |  |  |  |  |  |  |  |  |
|  | 41032 | Acute myocardial infarction of inferoposterior wall, subsequent episode of care |  |  |  |  |  |  |  |  |  |
|  | 41040 | Acute myocardial infarction of other inferior wall, episode of care unspecified |  |  |  |  |  |  |  |  |  |
|  | 41041 | Acute myocardial infarction of other inferior wall, initial episode of care |  |  |  |  |  |  |  |  |  |
|  | 41042 | Acute myocardial infarction of other inferior wall, subsequent episode of care |  |  |  |  |  |  |  |  |  |
|  | 41050 | Acute myocardial infarction of other lateral wall, episode of care unspecified |  |  |  |  |  |  |  |  |  |
|  | 41051 | Acute myocardial infarction of other lateral wall, initial episode of care |  |  |  |  |  |  |  |  |  |
|  | 41052 | Acute myocardial infarction of other lateral wall, subsequent episode of care |  |  |  |  |  |  |  |  |  |
|  | 41060 | True posterior wall infarction, episode of care unspecified |  |  |  |  |  |  |  |  |  |
|  | 41061 | True posterior wall infarction, initial episode of care |  |  |  |  |  |  |  |  |  |
|  | 41062 | True posterior wall infarction, subsequent episode of care |  |  |  |  |  |  |  |  |  |
|  | 41070 | Subendocardial infarction, episode of care unspecified |  |  |  |  |  |  |  |  |  |
|  | 41071 | Subendocardial infarction, initial episode of care |  |  |  |  |  |  |  |  |  |
|  | 41072 | Subendocardial infarction, subsequent episode of care |  |  |  |  |  |  |  |  |  |
|  | 41080 | Acute myocardial infarction of other specified sites, episode of care unspecified |  |  |  |  |  |  |  |  |  |
|  | 41081 | Acute myocardial infarction of other specified sites, initial episode of care |  |  |  |  |  |  |  |  |  |
|  | 41082 | Acute myocardial infarction of other specified sites, subsequent episode of care |  |  |  |  |  |  |  |  |  |
|  | 41090 | Acute myocardial infarction of unspecified site, episode of care unspecified |  |  |  |  |  |  |  |  |  |
|  | 41091 | Acute myocardial infarction of unspecified site, initial episode of care |  |  |  |  |  |  |  |  |  |
|  | 41092 | Acute myocardial infarction of unspecified site, subsequent episode of care |  |  |  |  |  |  |  |  |  |
|  | 4110 | Postmyocardial infarction syndrome |  |  |  |  |  |  |  |  |  |
|  | 4111 | Intermediate coronary syndrome |  |  |  |  |  |  |  |  |  |
|  | 41181 | Acute coronary occlusion without myocardial infarction |  |  |  |  |  |  |  |  |  |
|  | 41189 | Other acute and subacute forms of ischemic heart disease, other |  |  |  |  |  |  |  |  |  |
|  | 412 | Old myocardial infarction |  |  |  |  |  |  |  |  |  |
|  | 4130 | Angina decubitus |  |  |  |  |  |  |  |  |  |
|  | 4131 | Prinzmetal angina |  |  |  |  |  |  |  |  |  |
|  | 4139 | Other and unspecified angina pectoris |  |  |  |  |  |  |  |  |  |
|  | 41400 | Coronary atherosclerosis of unspecified type of vessel, native or graft |  |  |  |  |  |  |  |  |  |
|  | 41401 | Coronary atherosclerosis of native coronary artery |  |  |  |  |  |  |  |  |  |
|  | 41402 | Coronary atherosclerosis of autologous vein bypass graft |  |  |  |  |  |  |  |  |  |
|  | 41404 | Coronary atherosclerosis of artery bypass graft |  |  |  |  |  |  |  |  |  |
|  | 41405 | Coronary atherosclerosis of unspecified bypass graft |  |  |  |  |  |  |  |  |  |
|  | 41406 | Coronary atherosclerosis of native coronary artery of transplanted heart |  |  |  |  |  |  |  |  |  |
|  | 41407 | Coronary atherosclerosis of bypass graft (artery) (vein) of transplanted heart |  |  |  |  |  |  |  |  |  |
|  | 41410 | Aneurysm of heart (wall) |  |  |  |  |  |  |  |  |  |
|  | 41411 | Aneurysm of coronary vessels |  |  |  |  |  |  |  |  |  |
|  | 41412 | Dissection of coronary artery |  |  |  |  |  |  |  |  |  |
|  | 42090 | Acute pericarditis, unspecified |  |  |  |  |  |  |  |  |  |
|  | 42091 | Acute idiopathic pericarditis |  |  |  |  |  |  |  |  |  |
|  | 42099 | Other acute pericarditis |  |  |  |  |  |  |  |  |  |
|  | 4210 | Acute and subacute bacterial endocarditis |  |  |  |  |  |  |  |  |  |
|  | 4211 | Acute and subacute infective endocarditis in diseases classified elsewhere |  |  |  |  |  |  |  |  |  |
|  | 4219 | Acute endocarditis, unspecified |  |  |  |  |  |  |  |  |  |
|  | 4220 | Acute myocarditis in diseases classified elsewhere |  |  |  |  |  |  |  |  |  |
|  | 42290 | Acute myocarditis, unspecified |  |  |  |  |  |  |  |  |  |
|  | 42291 | Idiopathic myocarditis |  |  |  |  |  |  |  |  |  |
|  | 42292 | Septic myocarditis |  |  |  |  |  |  |  |  |  |
|  | 42293 | Toxic myocarditis |  |  |  |  |  |  |  |  |  |
|  | 42299 | Other acute myocarditis |  |  |  |  |  |  |  |  |  |
|  | 4230 | Hemopericardium |  |  |  |  |  |  |  |  |  |
|  | 4231 | Adhesive pericarditis |  |  |  |  |  |  |  |  |  |
|  | 4232 | Constrictive pericarditis |  |  |  |  |  |  |  |  |  |
|  | 4233 | Cardiac tamponade |  |  |  |  |  |  |  |  |  |
|  | 4238 | Other specified diseases of pericardium |  |  |  |  |  |  |  |  |  |
|  | 4240 | Mitral valve disorders |  |  |  |  |  |  |  |  |  |
|  | 4241 | Aortic valve disorders |  |  |  |  |  |  |  |  |  |
|  | 4242 | Tricuspid valve disorders, specified as nonrheumatic |  |  |  |  |  |  |  |  |  |
|  | 4243 | Pulmonary valve disorders |  |  |  |  |  |  |  |  |  |
|  | 42490 | Endocarditis, valve unspecified, unspecified cause |  |  |  |  |  |  |  |  |  |
|  | 42491 | Endocarditis in diseases classified elsewhere |  |  |  |  |  |  |  |  |  |
|  | 42499 | Other endocarditis, valve unspecified |  |  |  |  |  |  |  |  |  |
|  | 4250 | Endomyocardial fibrosis |  |  |  |  |  |  |  |  |  |
|  | 42511 | Hypertrophic obstructive cardiomyopathy |  |  |  |  |  |  |  |  |  |
|  | 42518 | Other hypertrophic cardiomyopathy |  |  |  |  |  |  |  |  |  |
|  | 4252 | Obscure cardiomyopathy of Africa |  |  |  |  |  |  |  |  |  |
|  | 4253 | Endocardial fibroelastosis |  |  |  |  |  |  |  |  |  |
|  | 4254 | Other primary cardiomyopathies |  |  |  |  |  |  |  |  |  |
|  | 4255 | Toxic myocarditis |  |  |  |  |  |  |  |  |  |
|  | 4257 | Nutritional and metabolic cardiomyopathy |  |  |  |  |  |  |  |  |  |
|  | 4258 | Cardiomyopathy in other diseases classified elsewhere |  |  |  |  |  |  |  |  |  |
|  | 4259 | Secondary cardiomyopathy, unspecified |  |  |  |  |  |  |  |  |  |
|  | 4260 | Atrioventricular block, complete |  |  |  |  |  |  |  |  |  |
|  | 42610 | Atrioventricular block, unspecified |  |  |  |  |  |  |  |  |  |
|  | 42611 | First degree atrioventricular block |  |  |  |  |  |  |  |  |  |
|  | 42612 | Mobitz (type) II atrioventricular block |  |  |  |  |  |  |  |  |  |
|  | 42613 | Other second degree atrioventricular block |  |  |  |  |  |  |  |  |  |
|  | 4262 | Left bundle branch hemiblock |  |  |  |  |  |  |  |  |  |
|  | 4263 | Other left bundle branch block |  |  |  |  |  |  |  |  |  |
|  | 4264 | Right bundle branch block |  |  |  |  |  |  |  |  |  |
|  | 42650 | Bundle branch block, unspecified |  |  |  |  |  |  |  |  |  |
|  | 42651 | Right bundle branch block and left posterior fascicular block |  |  |  |  |  |  |  |  |  |
|  | 42652 | Right bundle branch block and left anterior fascicular block |  |  |  |  |  |  |  |  |  |
|  | 42653 | Other bilateral bundle branch block |  |  |  |  |  |  |  |  |  |
|  | 42654 | Trifascicular block |  |  |  |  |  |  |  |  |  |
|  | 4266 | Other heart block |  |  |  |  |  |  |  |  |  |
|  | 4267 | Anomalous atrioventricular excitation |  |  |  |  |  |  |  |  |  |
|  | 42681 | Lown-Ganong-Levine syndrome |  |  |  |  |  |  |  |  |  |
|  | 42682 | Long QT syndrome |  |  |  |  |  |  |  |  |  |
|  | 42689 | Other specified conduction disorders |  |  |  |  |  |  |  |  |  |
|  | 4269 | Conduction disorder, unspecified |  |  |  |  |  |  |  |  |  |
|  | 4270 | Paroxysmal supraventricular tachycardia |  |  |  |  |  |  |  |  |  |
|  | 4271 | Paroxysmal ventricular tachycardia |  |  |  |  |  |  |  |  |  |
|  | 4272 | Paroxysmal tachycardia, unspecified |  |  |  |  |  |  |  |  |  |
|  | 42731 | Atrial fibrillation |  |  |  |  |  |  |  |  |  |
|  | 42732 | Atrial flutter |  |  |  |  |  |  |  |  |  |
|  | 42741 | Ventricular fibrillation |  |  |  |  |  |  |  |  |  |
|  | 42742 | Ventricular flutter |  |  |  |  |  |  |  |  |  |
|  | 4275 | Cardiac arrest |  |  |  |  |  |  |  |  |  |
|  | 42760 | Premature beats, unspecified |  |  |  |  |  |  |  |  |  |
|  | 42761 | Supraventricular premature beats |  |  |  |  |  |  |  |  |  |
|  | 42769 | Other premature beats |  |  |  |  |  |  |  |  |  |
|  | 42781 | Sinoatrial node dysfunction |  |  |  |  |  |  |  |  |  |
|  | 42789 | Other specified cardiac dysrhythmias |  |  |  |  |  |  |  |  |  |
|  | 4279 | Cardiac dysrhythmia, unspecified |  |  |  |  |  |  |  |  |  |
|  | 4280 | Congestive heart failure, unspecified |  |  |  |  |  |  |  |  |  |
|  | 4281 | Left heart failure |  |  |  |  |  |  |  |  |  |
|  | 42820 | Systolic heart failure, unspecified |  |  |  |  |  |  |  |  |  |
|  | 42821 | Acute systolic heart failure |  |  |  |  |  |  |  |  |  |
|  | 42822 | Chronic systolic heart failure |  |  |  |  |  |  |  |  |  |
|  | 42823 | Acute on chronic systolic heart failure |  |  |  |  |  |  |  |  |  |
|  | 42830 | Diastolic heart failure, unspecified |  |  |  |  |  |  |  |  |  |
|  | 42831 | Acute diastolic heart failure |  |  |  |  |  |  |  |  |  |
|  | 42832 | Chronic diastolic heart failure |  |  |  |  |  |  |  |  |  |
|  | 42833 | Acute on chronic diastolic heart failure |  |  |  |  |  |  |  |  |  |
|  | 42840 | Combined systolic and diastolic heart failure, unspecified |  |  |  |  |  |  |  |  |  |
|  | 42841 | Acute combined systolic and diastolic heart failure |  |  |  |  |  |  |  |  |  |
|  | 42842 | Chronic combined systolic and diastolic heart failure |  |  |  |  |  |  |  |  |  |
|  | 42843 | Acute on chronic combined systolic and diastolic heart failure |  |  |  |  |  |  |  |  |  |
|  | 4289 | Heart failure, unspecified |  |  |  |  |  |  |  |  |  |
|  | 4290 | Myocarditis, unspecified |  |  |  |  |  |  |  |  |  |
|  | 4291 | Myocardial degeneration |  |  |  |  |  |  |  |  |  |
|  | 4292 | Cardiovascular disease, unspecified |  |  |  |  |  |  |  |  |  |
|  | 4293 | Cardiomegaly |  |  |  |  |  |  |  |  |  |
|  | 4294 | Functional disturbances following cardiac surgery |  |  |  |  |  |  |  |  |  |
|  | 4295 | Rupture of chordae tendineae |  |  |  |  |  |  |  |  |  |
|  | 4296 | Rupture of papillary muscle |  |  |  |  |  |  |  |  |  |
|  | 42971 | Acquired cardiac septal defect |  |  |  |  |  |  |  |  |  |
|  | 42979 | Certain sequelae of myocardial infarction, not elsewhere classified, other |  |  |  |  |  |  |  |  |  |
|  | 42981 | Other disorders of papillary muscle |  |  |  |  |  |  |  |  |  |
|  | 42982 | Hyperkinetic heart disease |  |  |  |  |  |  |  |  |  |
|  | 42983 | Takotsubo syndrome |  |  |  |  |  |  |  |  |  |
|  | 42989 | Other ill-defined heart diseases |  |  |  |  |  |  |  |  |  |
|  | 4299 | Heart disease, unspecified |  |  |  |  |  |  |  |  |  |
|  | 3910 | Acute rheumatic pericarditis |  |  |  |  |  |  |  |  |  |
|  | 3911 | Acute rheumatic endocarditis |  |  |  |  |  |  |  |  |  |
|  | 3912 | Acute rheumatic myocarditis |  |  |  |  |  |  |  |  |  |
|  | 3918 | Other acute rheumatic heart disease |  |  |  |  |  |  |  |  |  |
|  | 3919 | Acute rheumatic heart disease, unspecified |  |  |  |  |  |  |  |  |  |
|  | 3920 | Rheumatic chorea with heart involvement |  |  |  |  |  |  |  |  |  |
|  | 3929 | Rheumatic chorea without mention of heart involvement |  |  |  |  |  |  |  |  |  |
|  | 393 | Chronic rheumatic pericarditis |  |  |  |  |  |  |  |  |  |
|  | 3940 | Mitral stenosis |  |  |  |  |  |  |  |  |  |
|  | 3941 | Rheumatic mitral insufficiency |  |  |  |  |  |  |  |  |  |
|  | 3942 | Mitral stenosis with insufficiency |  |  |  |  |  |  |  |  |  |
|  | 3949 | Other and unspecified mitral valve diseases |  |  |  |  |  |  |  |  |  |
|  | 3950 | Rheumatic aortic stenosis |  |  |  |  |  |  |  |  |  |
|  | 3951 | Rheumatic aortic insufficiency |  |  |  |  |  |  |  |  |  |
|  | 3952 | Rheumatic aortic stenosis with insufficiency |  |  |  |  |  |  |  |  |  |
|  | 3959 | Other and unspecified rheumatic aortic diseases |  |  |  |  |  |  |  |  |  |
|  | 3960 | Mitral valve stenosis and aortic valve stenosis |  |  |  |  |  |  |  |  |  |
|  | 3961 | Mitral valve stenosis and aortic valve insufficiency |  |  |  |  |  |  |  |  |  |
|  | 3962 | Mitral valve insufficiency and aortic valve stenosis |  |  |  |  |  |  |  |  |  |
|  | 3963 | Mitral valve insufficiency and aortic valve insufficiency |  |  |  |  |  |  |  |  |  |
|  | 3968 | Multiple involvement of mitral and aortic valves |  |  |  |  |  |  |  |  |  |
|  | 3969 | Mitral and aortic valve diseases, unspecified |  |  |  |  |  |  |  |  |  |
|  | 3970 | Diseases of tricuspid valve |  |  |  |  |  |  |  |  |  |
|  | 3971 | Rheumatic diseases of pulmonary valve |  |  |  |  |  |  |  |  |  |
|  | 3979 | Rheumatic diseases of endocardium, valve unspecified |  |  |  |  |  |  |  |  |  |
|  | 3980 | Rheumatic myocarditis |  |  |  |  |  |  |  |  |  |
| Chronic pulmonary disease |  |  |  |  |  |  |  |  |  |  |  |
|  | 4162 | Chronic pulmonary embolism |  |  |  |  |  |  |  |  |  |
|  | 4168 | Other chronic pulmonary heart diseases |  |  |  |  |  |  |  |  |  |
|  | 4169 | Chronic pulmonary heart disease, unspecified |  |  |  |  |  |  |  |  |  |
|  | 1144 | Tuberculous fibrosis of lung, tubercle bacilli not found (in sputum) by microscopy,  but found by bacterial culture |  |  |  |  |  |  |  |  |  |
|  | 4910 | Simple chronic bronchitis |  |  |  |  |  |  |  |  |  |
|  | 4911 | Mucopurulent chronic bronchitis |  |  |  |  |  |  |  |  |  |
|  | 49120 | Obstructive chronic bronchitis without exacerbation |  |  |  |  |  |  |  |  |  |
|  | 49121 | Obstructive chronic bronchitis with (acute) exacerbation |  |  |  |  |  |  |  |  |  |
|  | 49122 | Obstructive chronic bronchitis with acute bronchitis |  |  |  |  |  |  |  |  |  |
|  | 4918 | Other chronic bronchitis |  |  |  |  |  |  |  |  |  |
|  | 4919 | Unspecified chronic bronchitis |  |  |  |  |  |  |  |  |  |
|  | 49320 | Chronic obstructive asthma, unspecified |  |  |  |  |  |  |  |  |  |
|  | 49321 | Chronic obstructive asthma with status asthmaticus |  |  |  |  |  |  |  |  |  |
|  | 49322 | Chronic obstructive asthma with (acute) exacerbation |  |  |  |  |  |  |  |  |  |
|  | 496 | Chronic airway obstruction, not elsewhere classified |  |  |  |  |  |  |  |  |  |
|  | 500 | Coal workers' pneumoconiosis |  |  |  |  |  |  |  |  |  |
|  | 501 | Asbestosis |  |  |  |  |  |  |  |  |  |
|  | 504 | Pneumonopathy due to inhalation of other dust |  |  |  |  |  |  |  |  |  |
|  | 5064 | Chronic respiratory conditions due to fumes and vapors |  |  |  |  |  |  |  |  |  |
|  | 51283 | Chronic pneumothorax |  |  |  |  |  |  |  |  |  |
|  | 51883 | Chronic respiratory failure |  |  |  |  |  |  |  |  |  |
| Anemia |  |  |  |  |  |  |  |  |  |  |  |
|  | 2800 | Iron deficiency anemia secondary to blood loss (chronic) | | | | |  |  |  |  |  |
|  | 2808 | Other specified iron deficiency anemias | | |  |  |  |  |  |  |  |
|  | 2809 | Iron deficiency anemia, unspecified | | |  |  |  |  |  |  |  |
|  | 2810 | Pernicious anemia |  |  |  |  |  |  |  |  |  |
|  | 2811 | Other vitamin B12 deficiency anemia | | |  |  |  |  |  |  |  |
|  | 2812 | Folate-deficiency anemia | |  |  |  |  |  |  |  |  |
|  | 2818 | Anemia associated with other specified nutritional deficiency | | | | |  |  |  |  |  |
|  | 2819 | Unspecified deficiency anemia | |  |  |  |  |  |  |  |  |
|  | 2822 | Anemias due to disorders of glutathione metabolism | | | |  |  |  |  |  |  |
|  | 2829 | Hereditary hemolytic anemia, unspecified | | |  |  |  |  |  |  |  |
|  | 2830 | Autoimmune hemolytic anemias | |  |  |  |  |  |  |  |  |
|  | 2839 | Acquired hemolytic anemia, unspecified | | |  |  |  |  |  |  |  |
|  | 2849 | Aplastic anemia, unspecified | |  |  |  |  |  |  |  |  |
|  | 2851 | Acute posthemorrhagic anemia | |  |  |  |  |  |  |  |  |
|  | 2858 | Other specified anemias | |  |  |  |  |  |  |  |  |
|  | 2859 | Anemia, unspecified |  |  |  |  |  |  |  |  |  |
|  | 7765 | Congenital anemia |  |  |  |  |  |  |  |  |  |
|  | 7766 | Anemia of prematurity | |  |  |  |  |  |  |  |  |
|  | 28489 | Other specified aplastic anemias | |  |  |  |  |  |  |  |  |
|  | 28521 | Anemia in chronic kidney disease | | |  |  |  |  |  |  |  |
|  | 28522 | Anemia in neoplastic disease | |  |  |  |  |  |  |  |  |
|  | 28529 | Anemia of other chronic disease | |  |  |  |  |  |  |  |  |
|  | 5855 | Chronic kidney disease, Stage V | |  |  |  |  |  |  |  |  |
|  | 5856 | End stage renal disease | |  |  |  |  |  |  |  |  |
