# Supplementary material for: Development and Validation of a Clinical Prediction Model for Sleep Disorders in the ICU: A Retrospective Cohort Study
Source: Front Neurosci. 2021 Apr 16;15:644845. doi: 10.3389/fnins.2021.644845 (PMC8085546; doi:10.3389/fnins.2021.644845)
Supplement: Supplementary Material 4 — Patients with cognitive impairment from the MIMIC-III database according to ICD9-codes. [file Table_4.docx]

| **Supplementary material 4**  Patients with cognitive impairment from the MIMIC III database according to ICD9-codes | | |
| --- | --- | --- |
| Disease | ICD9-Code | Description |
| Sleep disorders |  |  |
|  | 29011 | Presenile dementia with delirium |
|  | 2903 | Senile dementia with delirium |
|  | 29041 | Vascular dementia, with delirium |
|  | 2910 | Alcohol withdrawal delirium |
|  | 29281 | Drug-induced delirium |
|  | 2930 | Delirium due to conditions classified elsewhere |
|  | 2931 | Subacute delirium |
|  | 30012 | Dissociative amnesia |
|  | 4377 | Transient global amnesia |
|  | 29283 | Drug-induced persisting amnestic disorder |
|  | 2911 | Alcohol-induced persisting amnestic disorder |
|  | 2940 | Amnestic disorder in conditions classified elsewhere |
|  | 2900 | Senile dementia, uncomplicated |
|  | 2900 | Senile dementia, uncomplicated |
|  | 29010 | Presenile dementia, uncomplicated |
|  | 29012 | Presenile dementia with delusional features |
|  | 29013 | Presenile dementia with depressive features |
|  | 29020 | Senile dementia with delusional features |
|  | 29021 | Senile dementia with depressive features |
|  | 29040 | Vascular dementia, uncomplicated |
|  | 29042 | Vascular dementia, with delusions |
|  | 29043 | Vascular dementia, with depressed mood |
|  | 2912 | Alcohol-induced persisting dementia |
|  | 29282 | Drug-induced persisting dementia |
|  | 29410 | Dementia in conditions classified elsewhere without behavioral disturbance |
|  | 29411 | Dementia in conditions classified elsewhere with behavioral disturbance |
|  | 29420 | Dementia, unspecified, without behavioral disturbance |
|  | 29421 | Dementia, unspecified, with behavioral disturbance |
|  | 33119 | Other frontotemporal dementia |
|  | 33182 | Dementia with lewy bodies |
|  | 3310 | Alzheimer's disease |
|  | 33183 | Mild cognitive impairment, so stated |
|  | 4380 | Late effects of cerebrovascular disease, cognitive deficits |
|  | 31400 | Attention deficit disorder without mention of hyperactivity |
|  | 31401 | Attention deficit disorder with hyperactivity |
|  | 79951 | Attention or concentration deficit |
|  | 79952 | Cognitive communication deficit |
|  | 79953 | Visuospatial deficit |
|  | 79959 | Other signs and symptoms involving cognition |
|  | 78093 | Memory loss |
