# Supplementary material for: Development and Validation of a Clinical Prediction Model for Sleep Disorders in the ICU: A Retrospective Cohort Study
Source: Front Neurosci. 2021 Apr 16;15:644845. doi: 10.3389/fnins.2021.644845 (PMC8085546; doi:10.3389/fnins.2021.644845)
Supplement: Supplementary Material 5 — Data Profiling Report. [file Table_5.pdf]

Data Profiling Report

- Basic Statistics
  - Raw Counts
  - Percentages
- Data Structure
- Missing Data Profile
- Univariate Distribution
  - Histogram
  - Bar Chart (by frequency)
  - QQ Plot
- Correlation Analysis
- Principal Component Analysis

Basic Statistics

Raw Counts

Name	Value
Rows	2,082
Columns	28
Discrete columns	1
Continuous columns	27
All missing columns	0
Missing observations	506
Complete Rows	1,681
Total observations	58,296
Memory allocation	427.4 Kb

Percentages

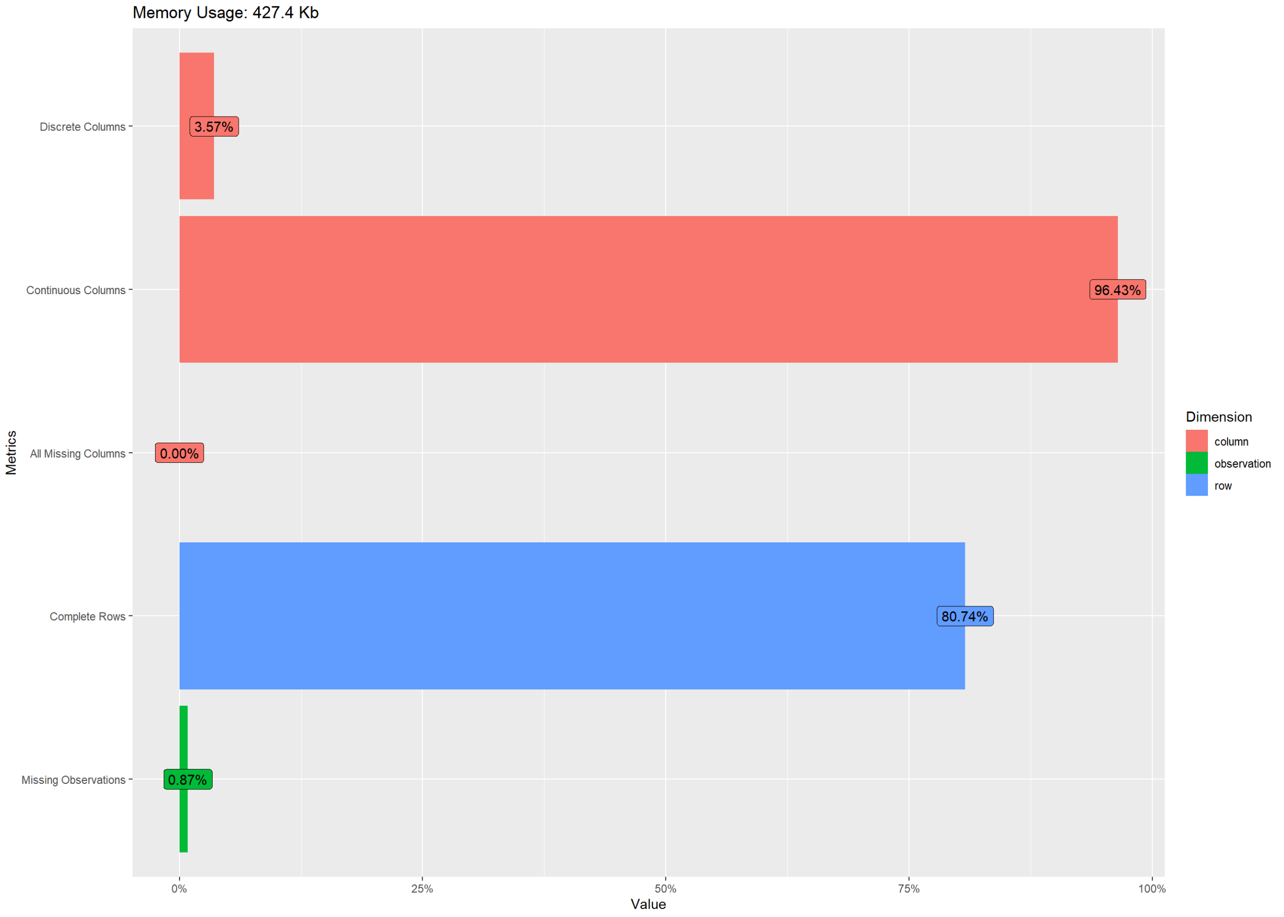

Data Structure

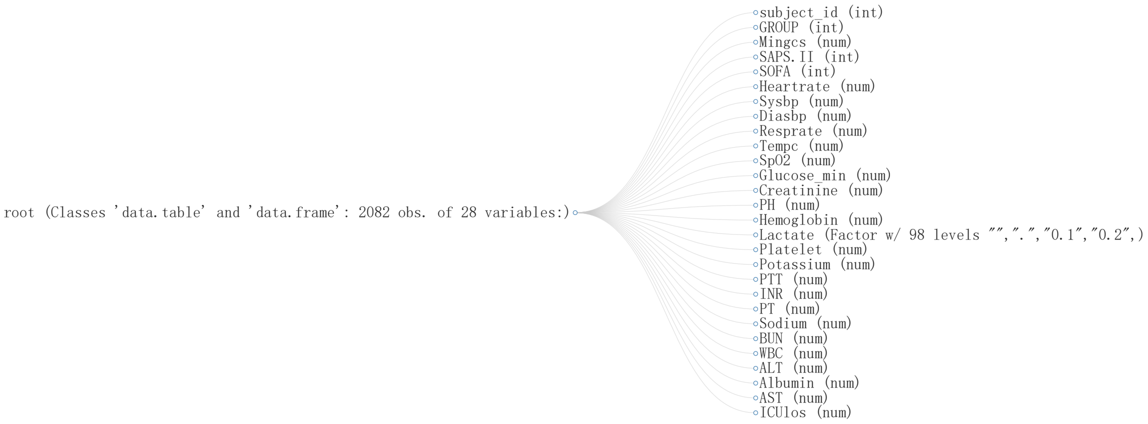

Missing Data Profile

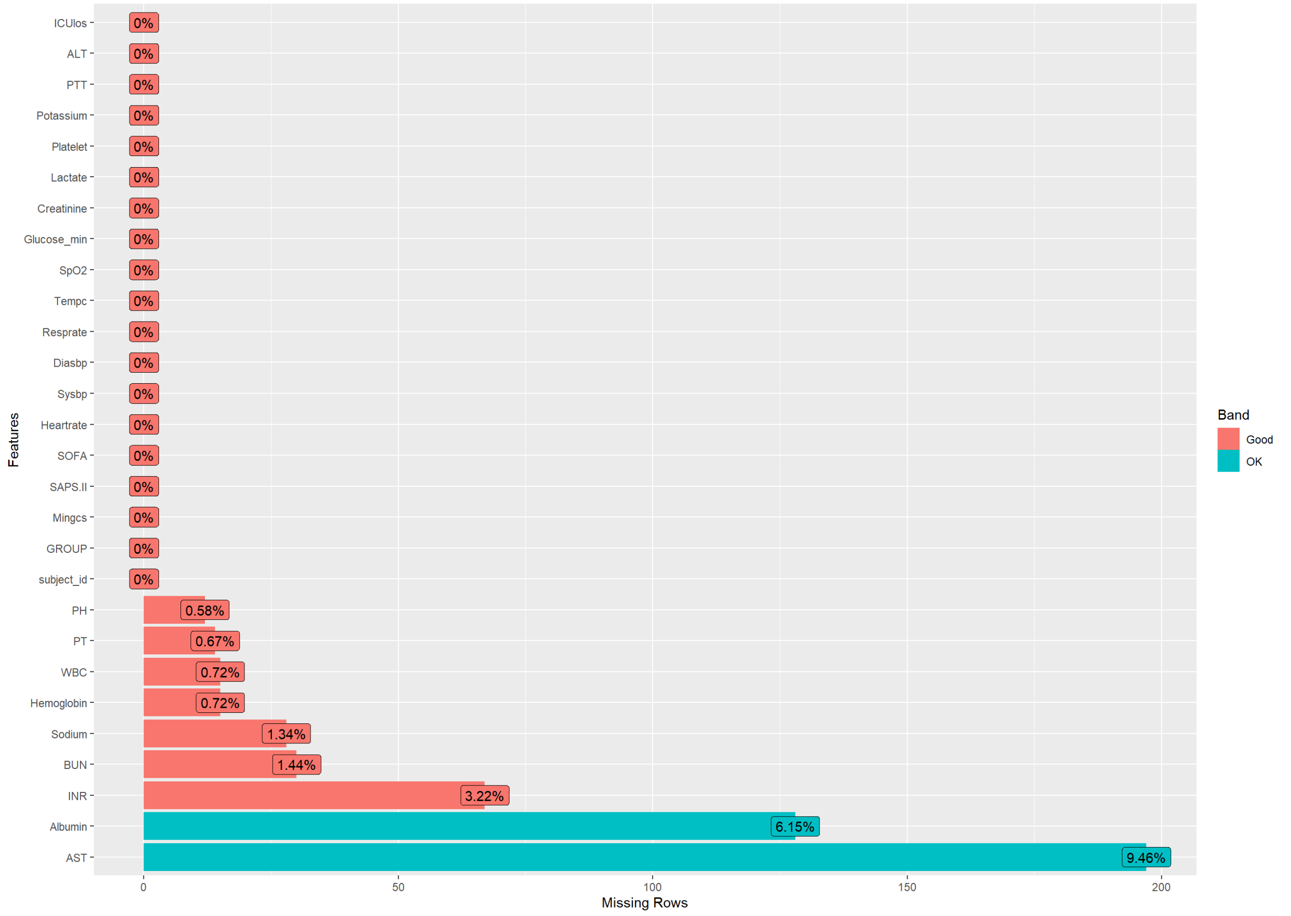

Univariate Distribution

Histogram

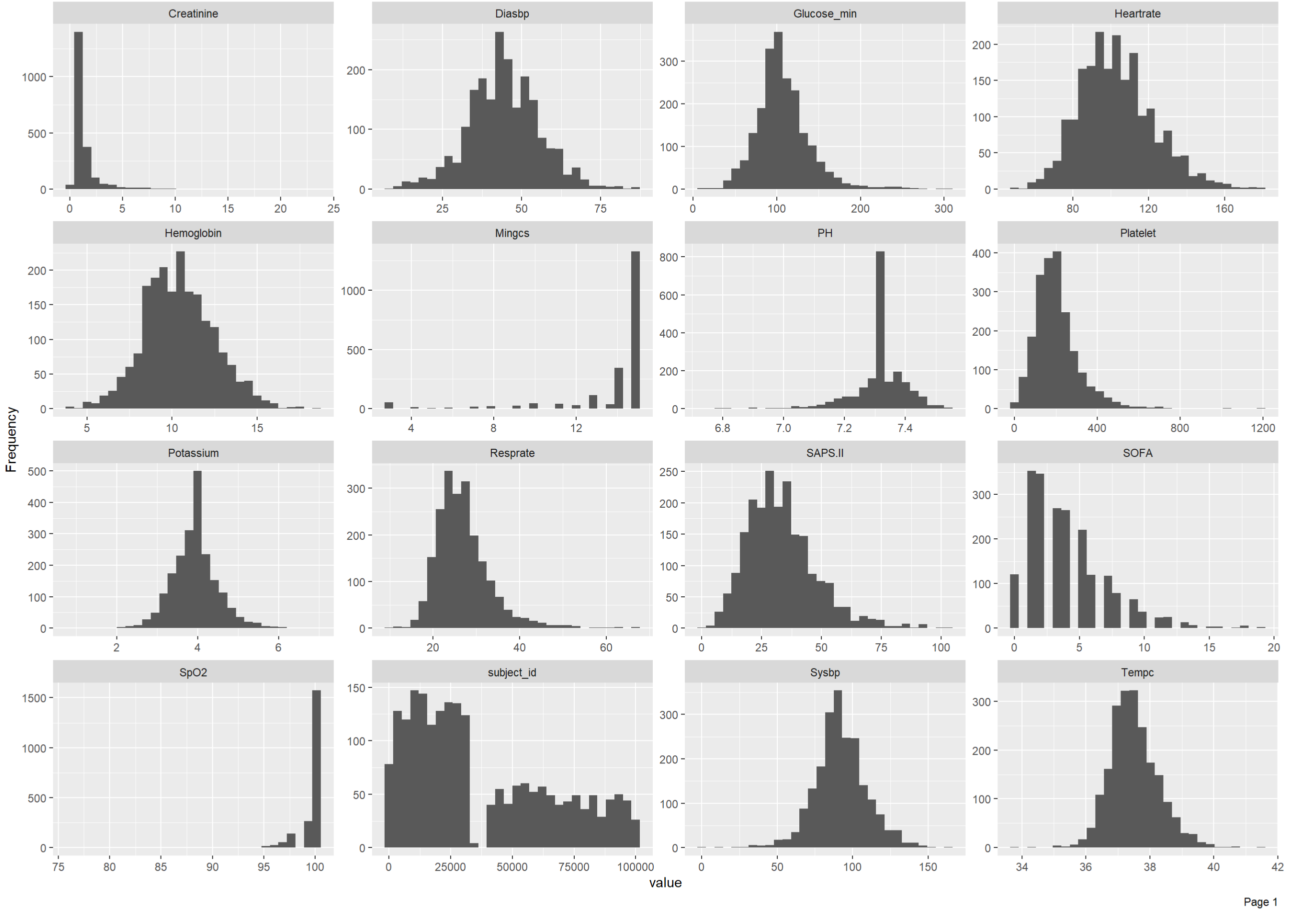

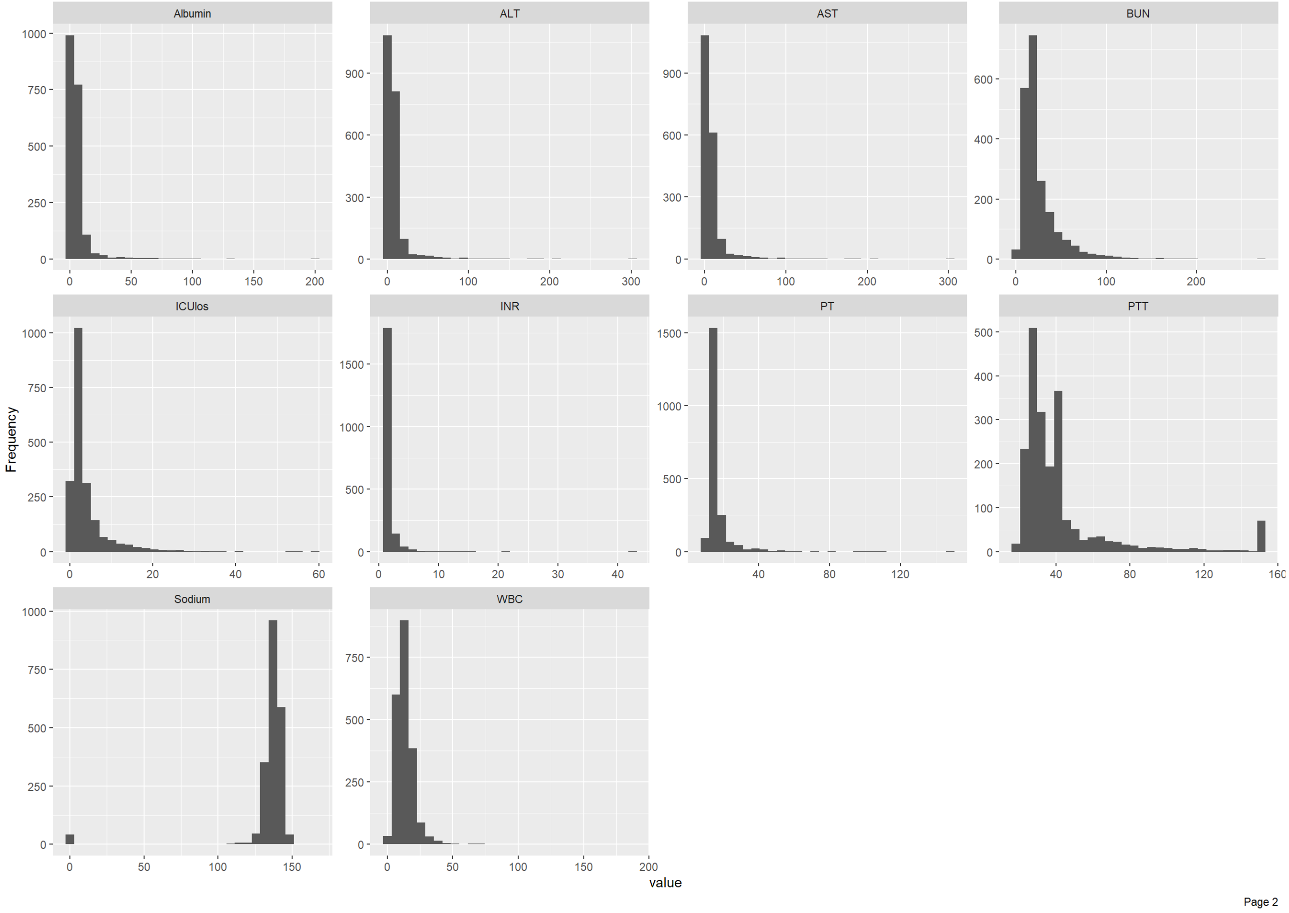

Bar Chart (by frequency)

```
## 1 columns ignored with more than 50 categories.
## Lactate: 98 categories
```

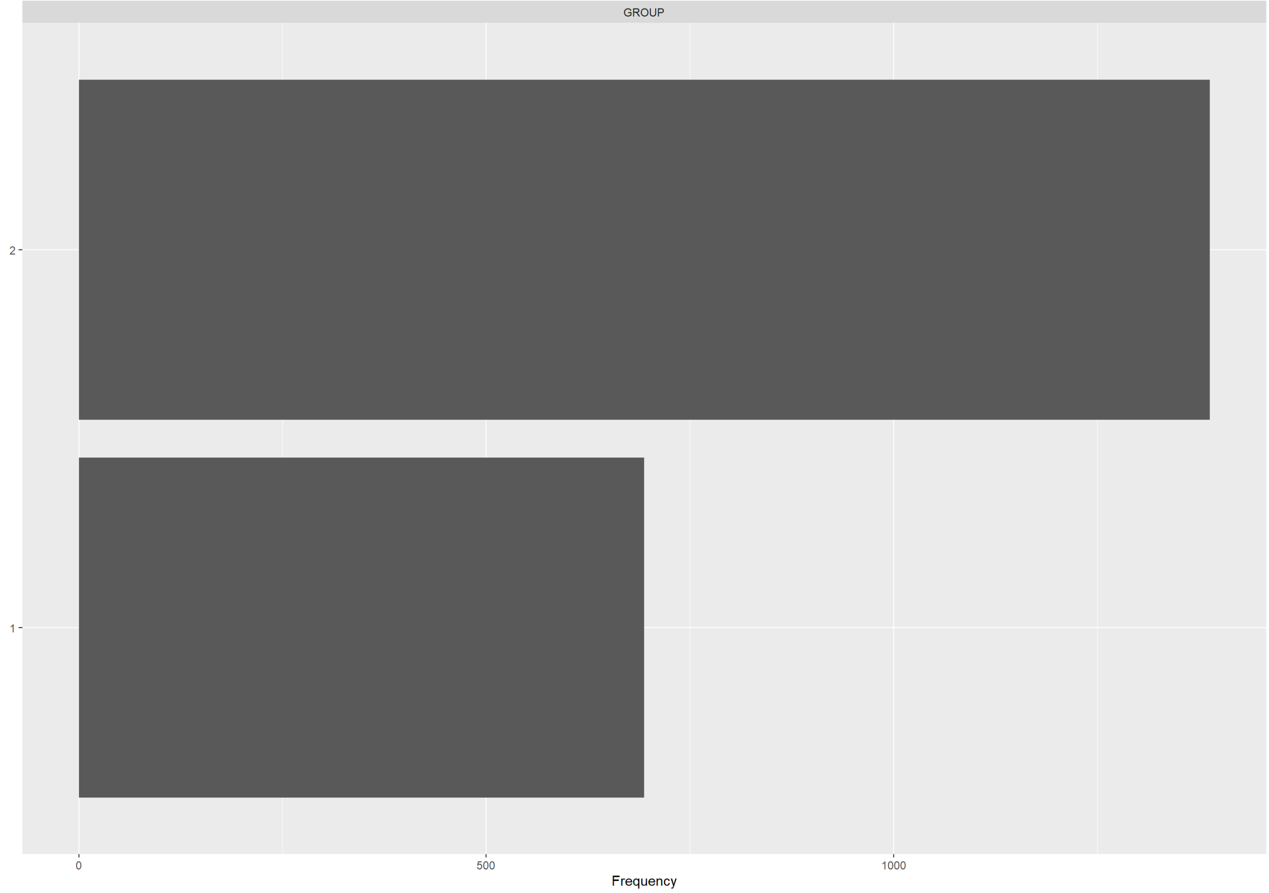

QQ Plot

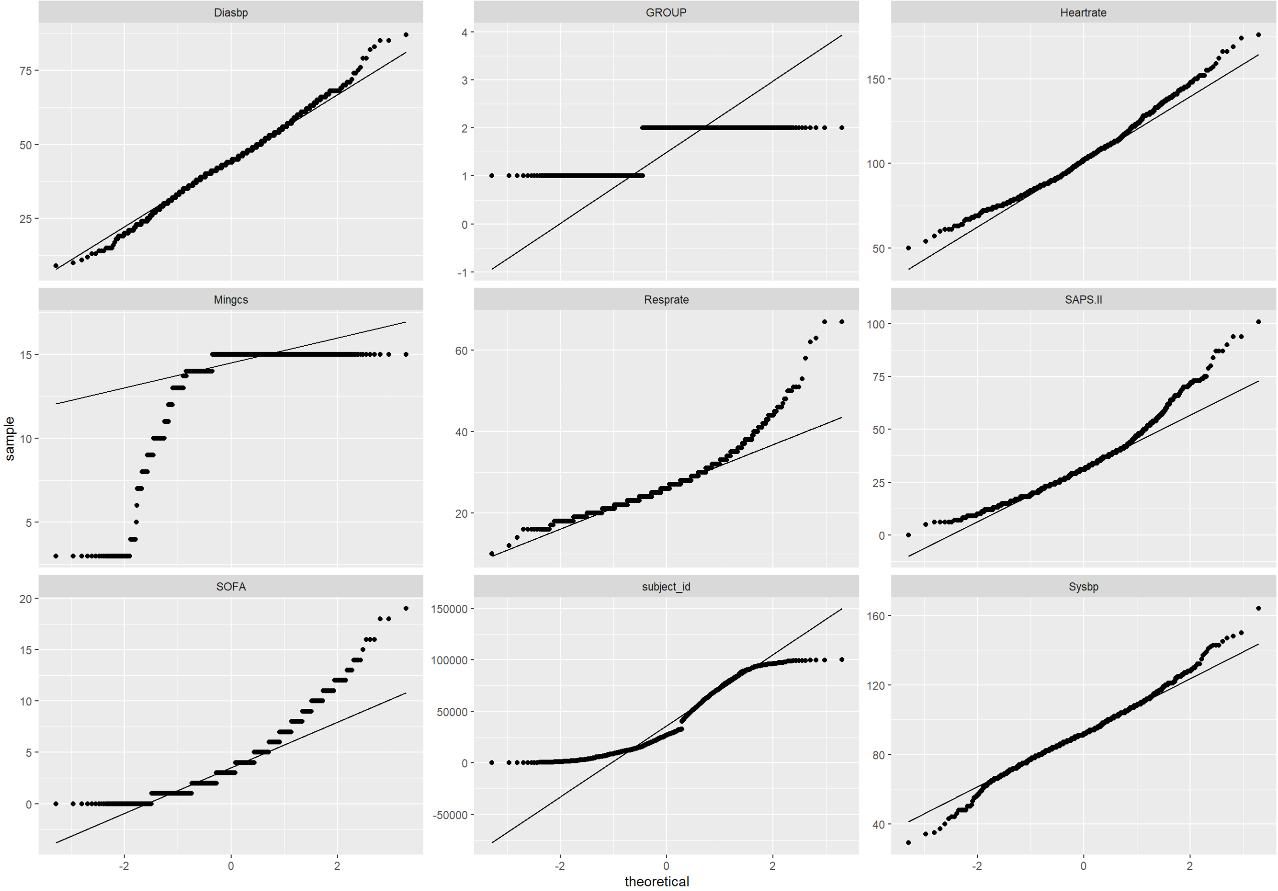
Diasbp
GROUP
Heartrate
Mingcs
Resprate
SAPS.II
SOFA
subject_id
Sysbp
sample
theoretical

Page 1

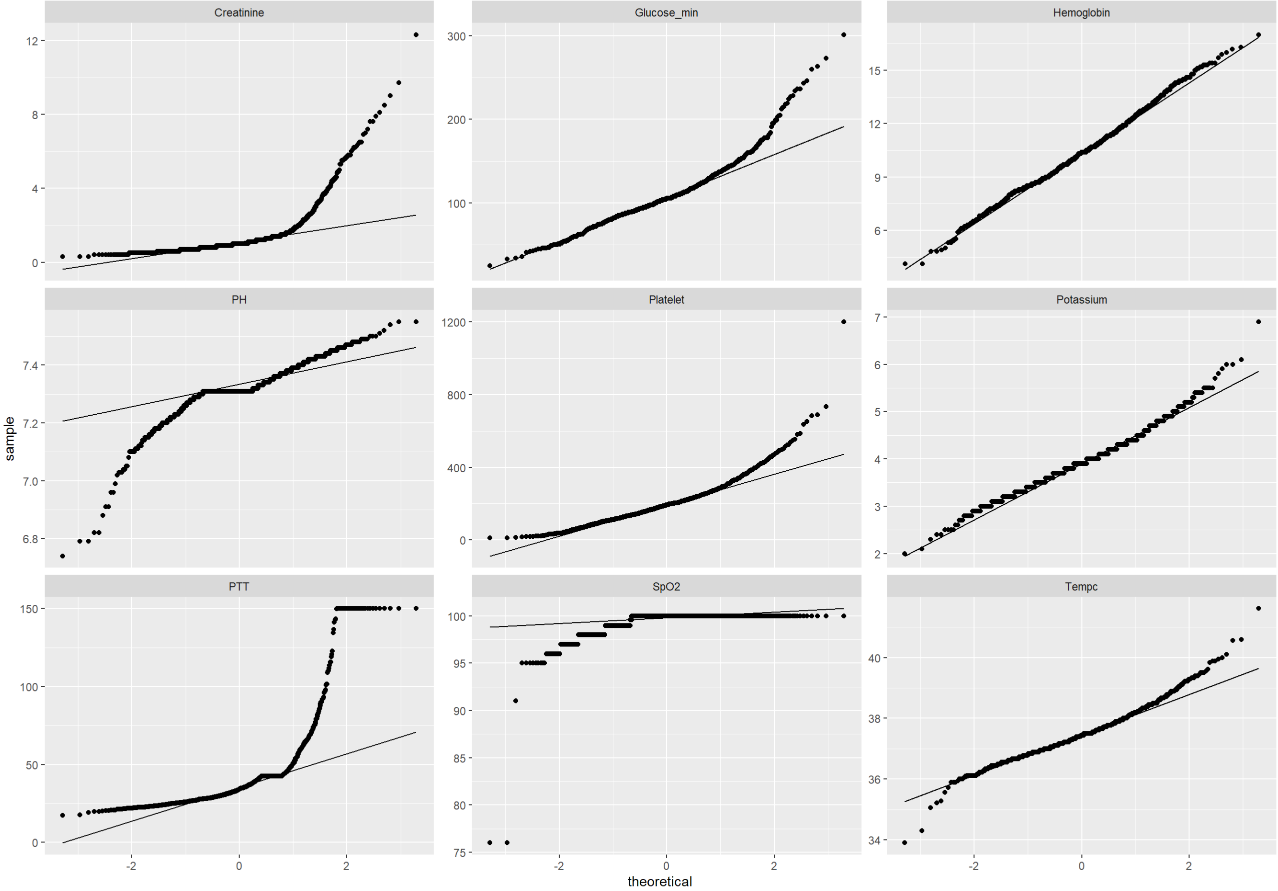
Creatinine
Glucose_min
Hemoglobin
PH
Platelet
Potassium
PTT
SpO2
Tempc
sample
theoretical

Page 2

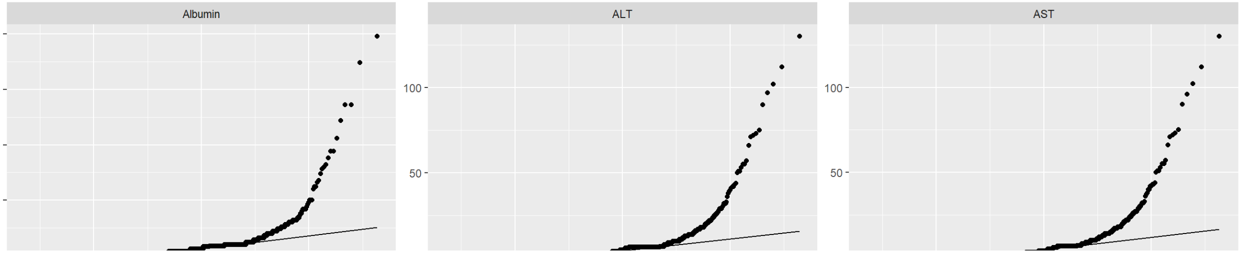
Albumin
ALT
AST

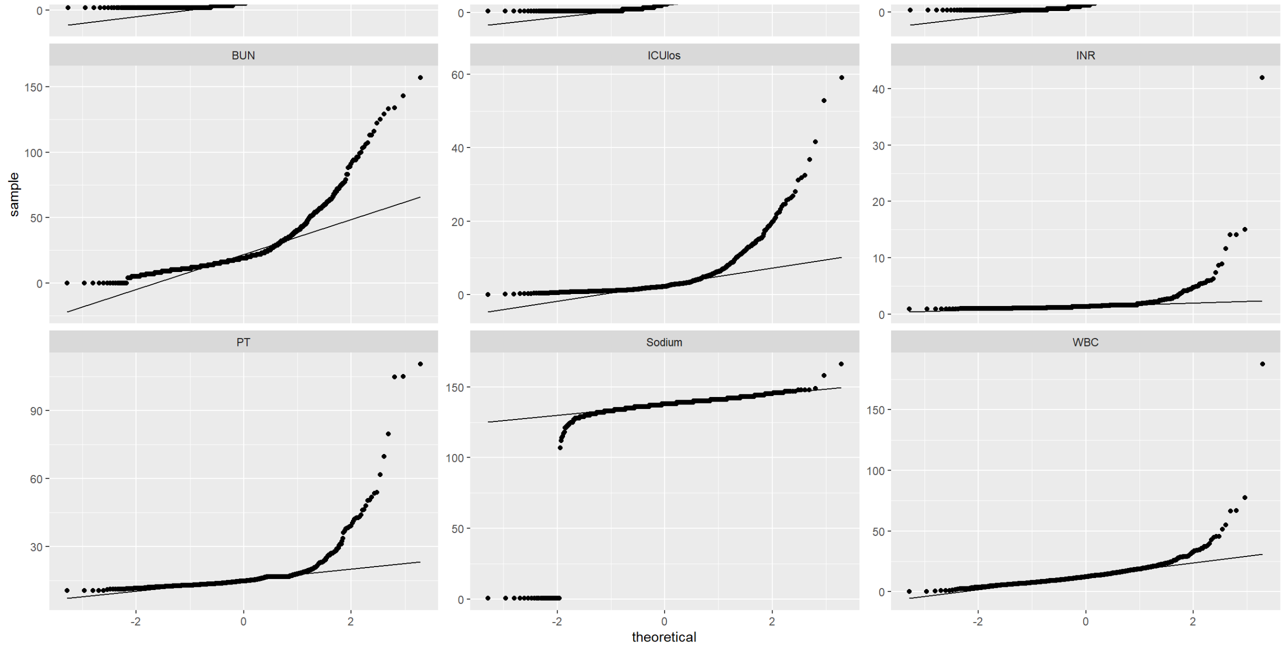

Correlation Analysis

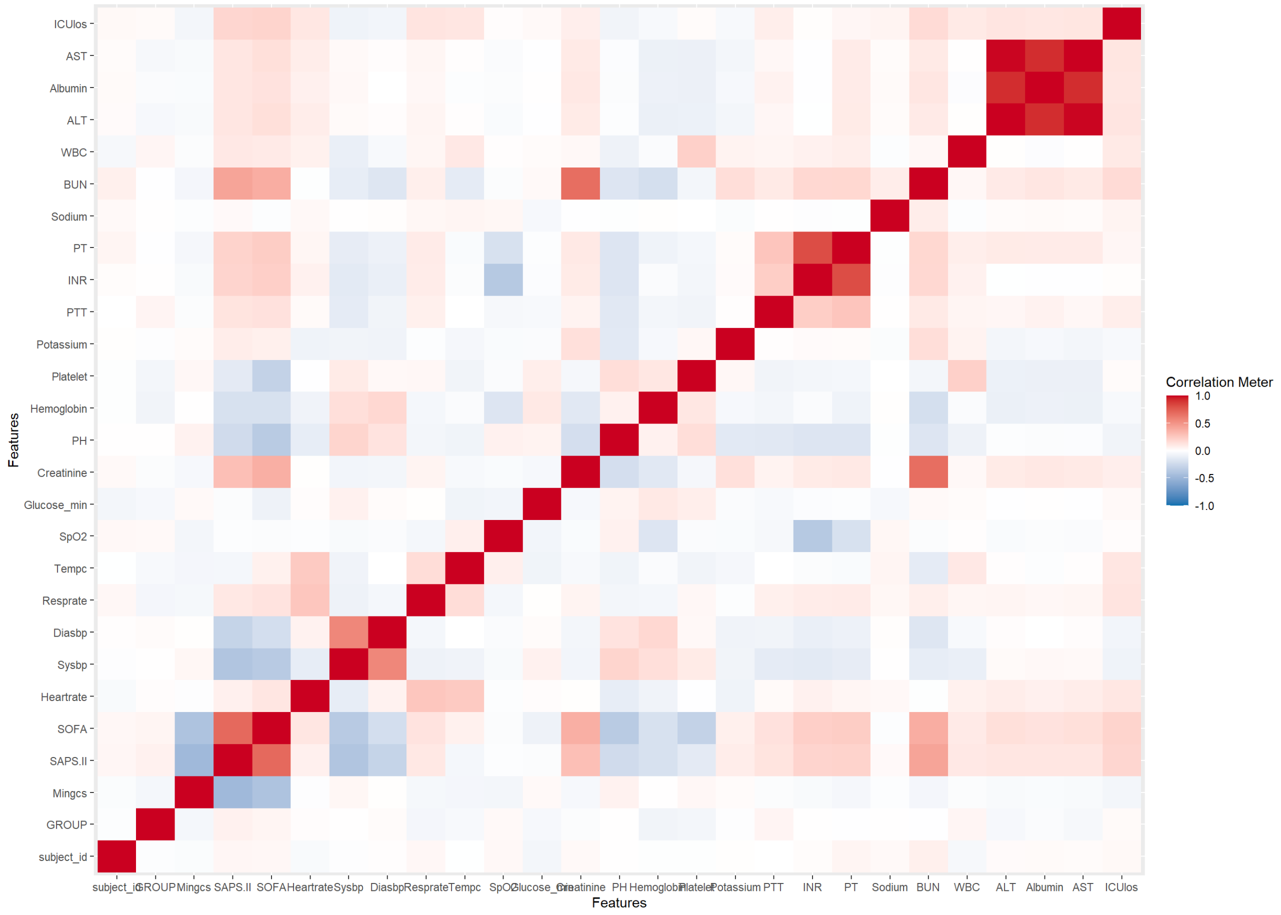

Principal Component Analysis

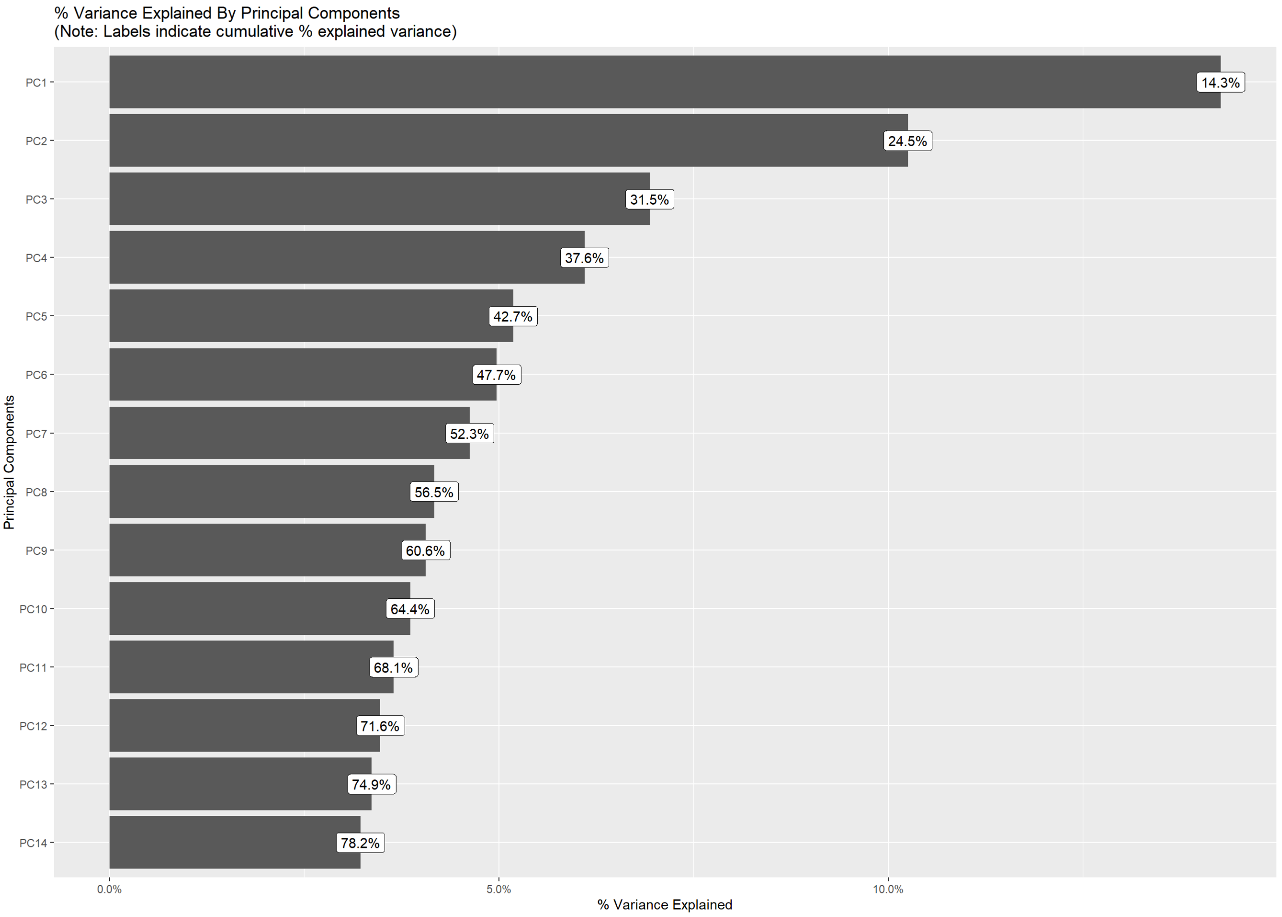
% Variance Explained By Principal Components
(Note: Labels indicate cumulative % explained variance)
Principal Components
PC1
PC2
PC3
PC4
PC5
PC6
PC7
PC8
PC9
PC10
PC11
PC12
PC13
PC14
14.3%
24.5%
31.5%
37.6%
42.7%
47.7%
52.3%
56.5%
60.6%
64.4%
68.1%
71.6%
74.9%
78.2%
0.0%
5.0%
10.0%
% Variance Explained

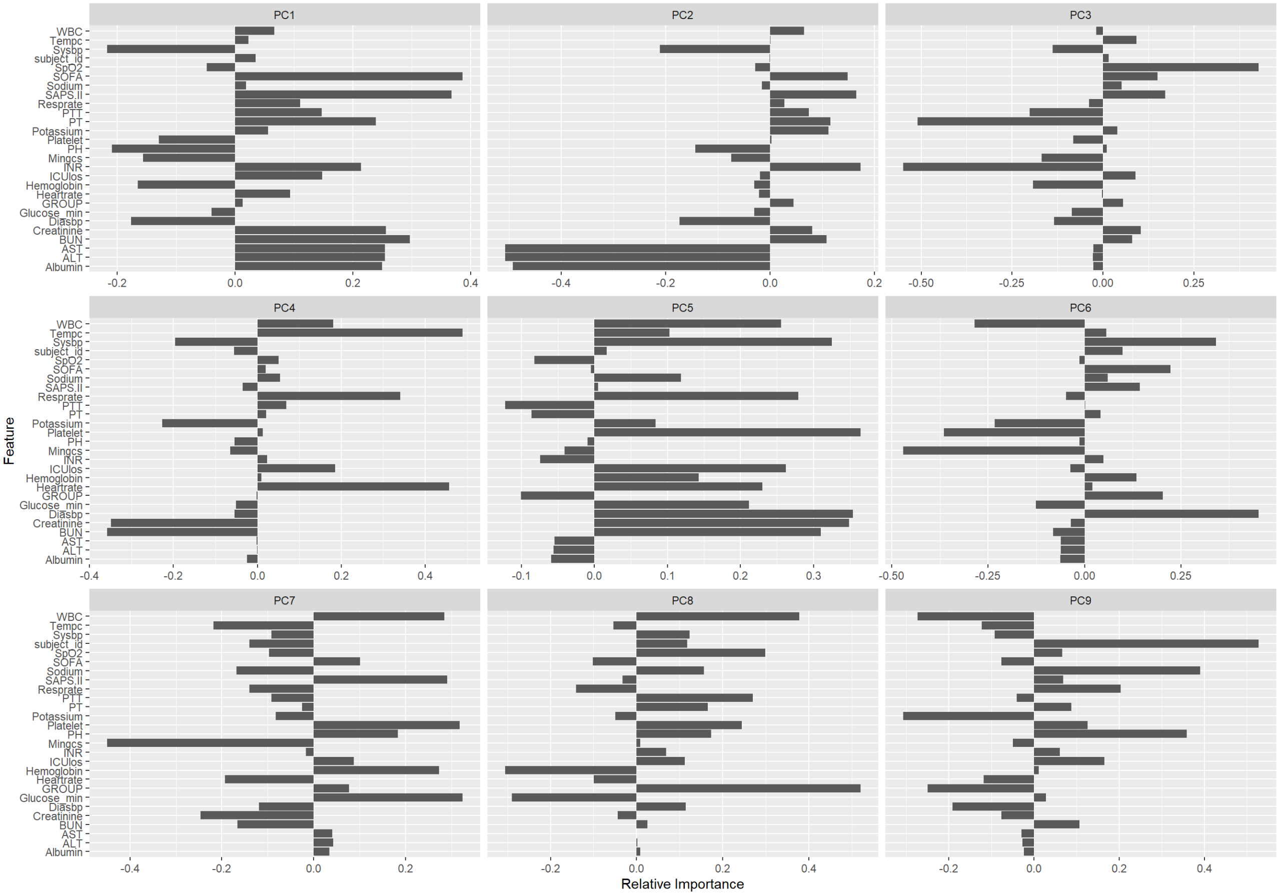
PC1
PC2
PC3
PC4
PC5
PC6
PC7
PC8
PC9
Feature
WBC
Tempc
Sysbp
subject_id
SpO2
SOFA
Sodium
SAPS.II
Resprate
PTT
PT
Potassium
Platelet
PH
Mingcs
INR
ICUlos
Hemoglobin
Heartrate
GROUP
Glucose_min
Diasbp
Creatinine
BUN
AST
ALT
Albumin
Relative Importance

Page 1

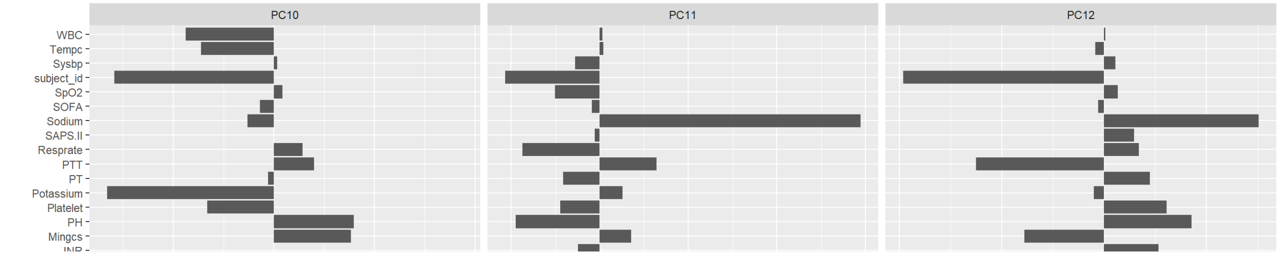
PC10
PC11
PC12
WBC
Tempc
Sysbp
subject_id
SpO2
SOFA
Sodium
SAPS.II
Resprate
PTT
PT
Potassium
Platelet
PH
Mingcs

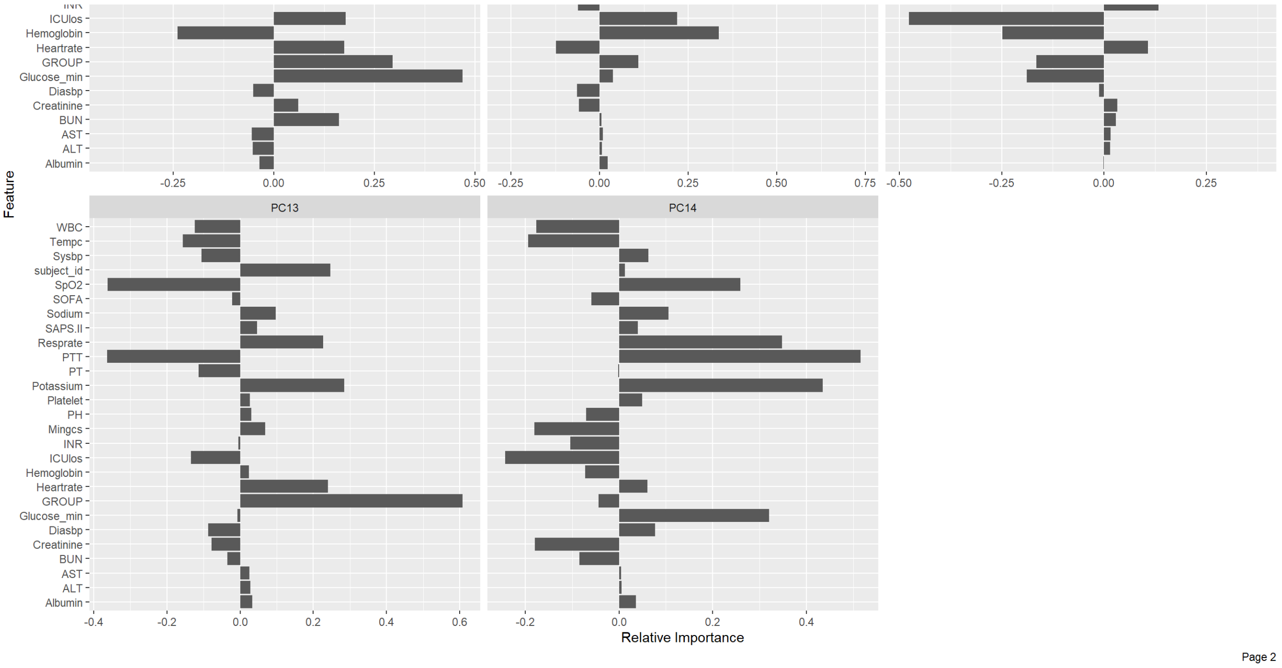
INR
ICUlos
Hemoglobin
Heartrate
GROUP
Glucose_min
Diasbp
Creatinine
BUN
AST
ALT
Albumin
Feature
PC13
PC14
WBC
Tempc
Sysbp
subject_id
SpO2
SOFA
Sodium
SAPS.II
Resprate
PTT
PT
Potassium
Platelet
PH
Mingcs
Relative Importance
Page 2
